# *Drosophila* Distal-less and Rotund Bind a Single Enhancer Ensuring Reliable and Robust *bric-a-brac2* Expression in Distinct Limb Morphogenetic Fields

**DOI:** 10.1371/journal.pgen.1003581

**Published:** 2013-06-27

**Authors:** Aissette Baanannou, Luis Humberto Mojica-Vazquez, Gaylord Darras, Jean-Louis Couderc, David L. Cribbs, Muriel Boube, Henri-Marc Bourbon

**Affiliations:** 1 Centre de Biologie du développement, UMR5547 CNRS/UPS, Université de Toulouse, Toulouse, France; 2 Laboratoire Génétique, Reproduction et Développement, UMR6293, CNRS/Université de Clermont Ferrand, Clermont-Ferrand, France; University of Dayton, United States of America

## Abstract

Most identified *Drosophila* appendage-patterning genes encode DNA-binding proteins, whose cross-regulatory interactions remain to be better characterized at the molecular level, notably by studying their direct binding to tissue-specific transcriptional enhancers. A fine-tuned spatio-temporal expression of *bric-a-brac2* (*bab2*) along concentric rings is essential for proper proximo-distal (P-D) differentiation of legs and antennae. However, within the genetic interaction landscape governing limb development, no transcription factor directly controlling *bab2* expression has been identified to date. Using site-targeted GFP reporter assay and BAC recombineering, we show here that restricted *bab2* expression in leg and antennal imaginal discs relies on a single 567-bp-long *cis*-regulatory module (CRM), termed LAE (for leg and antennal enhancer). We show that this CRM (i) is necessary and sufficient to ensure normal *bab2* activity in developing leg and antenna, and (ii) is structurally and functionally conserved among Drosophilidae. Through deletion and site-directed mutagenesis approaches, we identified within the LAE essential sequence motifs required in both leg and antennal tissues. Using genetic and biochemical tests, we establish that in the LAE (i) a key TAAT-rich activator motif interacts with the homeodomain P-D protein Distal-less (Dll) and (ii) a single T-rich activator motif binds the C2H2 zinc-finger P-D protein Rotund (Rn), leading to *bab2* up-regulation respectively in all or specifically in the proximal-most ring(s), both in leg and antenna. Joint ectopic expression of Dll and Rn is sufficient to cell-autonomously activate endogenous *bab2* and LAE-driven reporter expression in wing and haltere cells. Our findings indicate that accuracy, reliability and robustness of developmental gene expression do not necessarily require *cis*-regulatory information redundancy.

## Author Summary

In insects, leg and antenna are homologous limbs, though derive from a single ancestral appendage. In *Drosophila*, leg and antennal development along the proximo-distal (P-D) axis relies on relatively-well known genetic cascades, in which most appendage-patterning genes encode transcription factors (TF). However, their cross-regulatory interactions remain to be better characterized at the molecular level. A fine-tuned expression of the *bric-a-brac2* (*bab2*) gene is essential for normal leg and antennal segmentation. However, within the genetic cascades governing P-D limb development, no TF directly controlling *bab2* expression has been identified to date. We show here that restricted *bab2* expression in developing leg and antenna is governed by a single enhancer, termed LAE, which is necessary and sufficient *in-vivo* to ensure *bab2* functions there. We show that leg and antennal *cis*-regulatory elements are closely associated and that essential LAE sites interact with Distal-less (Dll) and Rotund (Rn) TFs, leading to *bab2* activation in all or specifically in the proximal-most expressing cells, respectively. Finally, joint ectopic expression of Dll and Rn is sufficient to instruct wing and haltere cells to up-regulate *bab2*. Taken together, our work indicates that a single enhancer is necessary and sufficient to reliably govern *bab2* expression in distinct morphogenetic fields.

## Introduction

Fine-tuned spatial and temporal transcriptional regulation is essential to ensure proper development [1], [2]. Reliability of developmental gene regulation is governed by tissue-specific *cis*-regulatory modules (CRM) or “enhancers”, often situated far away from gene promoters, whereas robustness and accuracy of gene expression could be ascribed to partially-redundant “shadow” enhancers [3]. Morphogenesis of the *Drosophila* adult leg along the proximo-distal (P-D) axis offers a good model to decipher how patterning genes are tightly controlled at the transcriptional level and integrated within the well characterized limb-specific genetic cascades [4]–[9]. Nevertheless, owing to the large number of leg patterning genes encoding DNA-binding proteins and the complexity of their cross interactions, it is a challenge to determine which transcription factors (TF) are directly implicated in the regulation of a given P-D gene and how they functionally interact upon binding to discrete DNA sites. Toward a full understanding at the molecular level of the genetic interaction landscape governing limb development, it is thus crucial to gain better knowledge of the CRM(s) controlling the fine-tuned expression of each member of the underlying gene regulatory network.

The *Drosophila* leg is composed of ten segments which are articulated to each other by characteristic joints [8]. The distal portion of the adult leg, the tarsus, is divided into five segments (ts1–5), and P-D tarsal patterning occurs by successive intercalations of new positional fates within the growing leg imaginal disc [5], [9]. Early on during this process, *wingless* and *decapentaplegic* signalling pathways regulate the expression of transcription factors encoded by *Distal-less* (*Dll*), *dachshund* (*dac*) and *homothorax* (*hth*) P-D genes [10]–[13]. *Dll*, *dac* and *hth* are activated in concentric domains that define the distal, medial and proximal parts of the adult appendages, respectively [14], [15]. Together with EGFR signalling emanating from the distal-most cells, these so-called leg “gap” genes in turn regulate downstream TF-encoding genes, that include *spineless*, *rotund*, *bric-a-brac1/2*, *BarH1/*2 and *apterous*, all expressed dynamically in the tarsal domain [5], [9]. Unlike the leg, in the antennal imaginal disc *Dll* and *hth* expression domains overlap, leading to the specific activation of *spalt* and *cut* [16]. Except for the regulation of the early-acting *Dll* and *dac* gap genes [14], [15], few direct interactions have been established within the leg and antennal P-D regulatory cascades. Here, we choose the *bric-a-brac* (*bab*) locus as a model to study the integrated regulation of P-D patterning genes implicated in distal leg and antennal segmentation.

The *bab* locus consists of two paralogous genes, *bab1* and *bab2*, encoding BTB transcription factors [17], [18]. Although both genes are partially redundant in other developmental processes, only *bab2* is indispensible for distal leg and antennal segmentation [17], [18]. However, both *bab1* and *bab2* display dynamic expression in similar restricted P-D sub-domains with distinct expression patterns between leg and antenna [17], [18]. Initially expressed homogeneously within the *Dll*-expressing distal domain in early-mid third-instar larvae (L3), the *bab1*/*bab2* expression pattern in late L3 resolves to four concentric rings in the leg or two concentric rings in the antennal imaginal discs [17]. Later on at pupal and adult stages a P-D expression gradient within each ring is observed which is essential for ts2–4 and antennal a3–5 segment joint formation [17]–[19].

In both developing leg and antenna, all aspects of the *bab2* expression pattern require the activity of the homeodomain Dll protein [19]–[21]. In addition to *Dll*, *bab2* expression is dependent on *spineless* (*ss*), at the exclusion of the distal-most ring [19]. Indeed, *spineless* encodes a bHLH-PAS family TF that is transiently expressed in ts1–3 from early to mid L3 stage [19], [21], [22]. Of note, unlike leg, *ss* expression is maintained in developing antenna [22], notably under the direct control of Dll [23]. In addition to positive inputs from *Dll* and *ss*, *bab2* expression is restricted proximally by *dac* activity [19] and distally by a gradient of EGFR signalling [21], [24]. Lastly, graded *bab2* expression, both distally and proximally, has been linked to Notch signalling via a repressive effect of *bowl* activity [25], [26].

Although several *bab2* regulators have been identified, to date none has been shown to be direct and no limb-specific CRM has been identified. Starting from a previous systematic identification of tissue-specific enhancers within the 150-kilobase (kb)-long *bab* locus performed by Williams et al. [27], we characterized here an evolutionarily-conserved 567 base pair (bp) CRM, which reproduces expression of the *bab2* endogenous gene, both in leg and antennal tissues. This CRM (termed LAE, for leg and antennal enhancer) is physically and functionally conserved between *D. melanogaster* and *D. virilis*. We find that the LAE is both necessary and sufficient *in-vivo* to ensure proper *bab2* expression in leg and antennal imaginal discs, and for normal segmentation of the mature appendices. Using targeted deletions and site-directed mutagenesis, we show that leg and antennal *cis*-regulatory elements are closely associated. Furthermore, activation of *bab2* expression in proximal- and distal-most rings is dependent on separate DNA elements. Moreover, we show that discrete essential LAE sites interact with Distal-less and Rotund transcription factors leading to *bab2* activation in all or specifically in the proximal-most expressing cells, respectively. Finally, ectopic co-expression of Dll and Rn is sufficient to instruct wing and haltere cells to up-regulate *bab2*. Taken together, our work indicates that a single enhancer, under the direct control of the P-D proteins Dll and Rn, is necessary and sufficient to reliably govern *Drosophila bab2* expression in distinct limb morphogenetic fields.

## Results

### A conserved *cis*-regulatory module recapitulates limb-specific *bab2* expression

**Figure 1:**
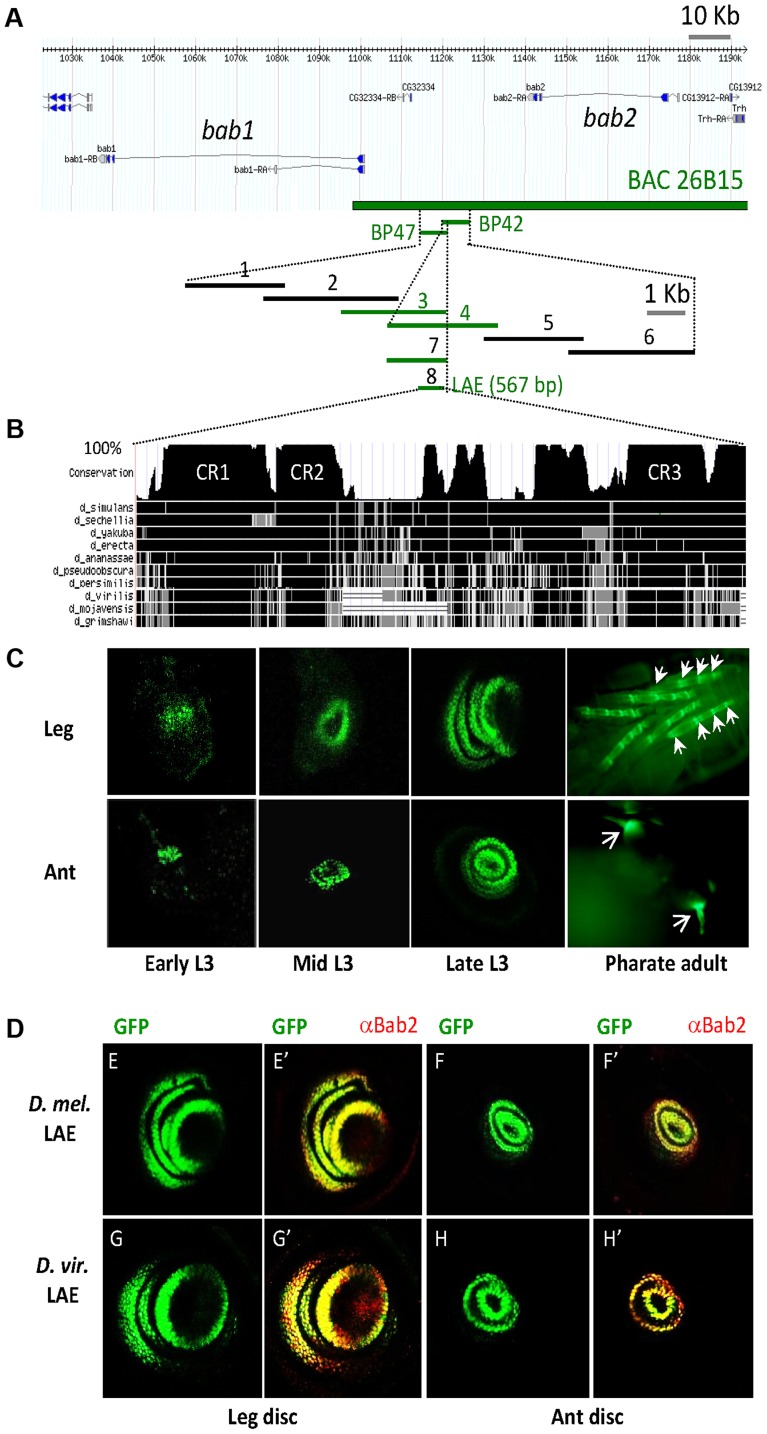
Identification of an evolutionarily-conserved CRM that recapitulates leg and antennal *bab2*-expression. (A) A GFP-reporter assay identified a leg/antennal enhancer (LAE) element situated in the middle of the 150-kb *bric-a-brac* locus, between the *bab1* and *bab2* transcription units. The position of the 26B15 BAC used for phenotypic rescue experiments is shown, as well as the two overlapping genomic fragments (BP42 and BP47) driving GFP-reporter expression in developing legs [27]. Beneath, an enlarged view of the 11-kb BP42-47 region shows the positions of the eight genomic sub-fragments (#1–8) tested in our site-directed GFP reporter assay (see Materials and methods). The four fragments (#3, 4, 7 and 8) that faithfully reproduce wild-type leg and antennal (ant) *bab2* expression (Figure S1) are indicated in green. (B) Conservation of the LAE sequence among Drosophilidae. Identical, highly- or non-conserved LAE sequences among a set of 10 *Drosophila* species, with respect to the *D. melanogaster* sequence, are depicted, respectively, by thick vertical black, grey or white lines, whereas thin horizontal grey lines indicate gaps. As determined from the UCSC genome browser (http://genome.ucsc.edu/), sequence conservation (from 0 to 100% identity) over a 10-bp sliding window is represented above by the height of the conservation plot, with the three >20-pb-long highly-conserved CR1-3 regions indicated within (A full alignment from LAE sequences of 22 Drosophilidae species is provided in Supplementary Figure S2). (C) The 567 bp LAE recapitulates spatial and temporal *bab2*-expression pattern in developing leg and antenna. The expression of the *LAE-GFP* reporter construct is shown for early, mid and late third-instar larval as well as pharate adult stages. Expression sites in mature appendages are indicated by arrows. (D) Functional conservation of the *D. virilis* LAE regulatory activity in developing *D. melanogaster* appendages. GFP expression (green) alone and in combination with Bab2 immunostaining (red) of leg (E–E′ and G–G′, respectively) and antennal (F–F′ and H–H′, respectively) imaginal discs from late third-instar larvae expressing either the *D. melanogaster* (*D. mel*) or *D. virilis* (*D. vir.*) *LAE-GFP* construct (E–F and G–H, respectively), are shown.

A systematic study of the 150-kb *bab* locus identified leg-specific *cis*-regulatory elements within a 11 kb region encompassing two overlapping genomic fragments (termed BP42 and BP47) localized between the *bab1* and *bab2* transcription units (Figure 1A) [27]. Both BP42 and BP47 fragments also reproduce the antennal *bab2* expression (Supplementary Figure S1). To identify limb-specific *bab* CRMs, we further dissected the relevant 11-kb region, using a sequence-directed GFP reporter assay (see Materials and methods) [28]. Six overlapping genomic fragments (#1 to 6) (Figure 1A) were examined for GFP expression in both developing leg and antenna. Only fragments #3 and #4 drove strong GFP expression in leg as well as antennal tissues (Figure S1) indicating that the relevant cis-regulatory information is located within the 1.5 kb sequence shared by BP42 and BP47. In confirmation of this, a fragment (#7) containing only this 1.5 kb region was sufficient to reliably reproduce *bab2* expression in leg and antennal tissues (Figure S1).

Before dissecting further the 1.5 kb fragment, we examined its evolutionary conservation among 12 *Drosophila* species whose genome sequences were available [29]. LAE sequences were identified and aligned. Only three >20 bp motifs (termed CR1-3) are highly conserved among Drosophilidae (Figures 1B and S2). We therefore tested the activity of a 567-bp fragment (#8) encompassing the CR1-3 motifs, and found that its activity faithfully recapitulated spatial and temporal *bab2* expression in both developing leg and antenna (Figure 1C–D). From two Drosophilidae species having diverged 40–60 millions years ago, we then tested the equivalent 0.7-kb *D. virilis* region and found that its regulatory activity was similar to that of the *D. melanogaster* LAE (Figure 1D, compare G–G′ and H–H′ to E–E′ and F–F′, respectively), supporting the functional importance of the conserved CR1-3 motifs. Taken together these data indicate that the evolutionarily-conserved 567-bp region contains regulatory information sufficient to recapitulate limb-specific *bab2* expression, both in developing leg and antenna. We therefore termed this region LAE, for leg and antennal enhancer.

### LAE is necessary and sufficient for *bab2* expression and function *in-vivo*

**Figure 2:**
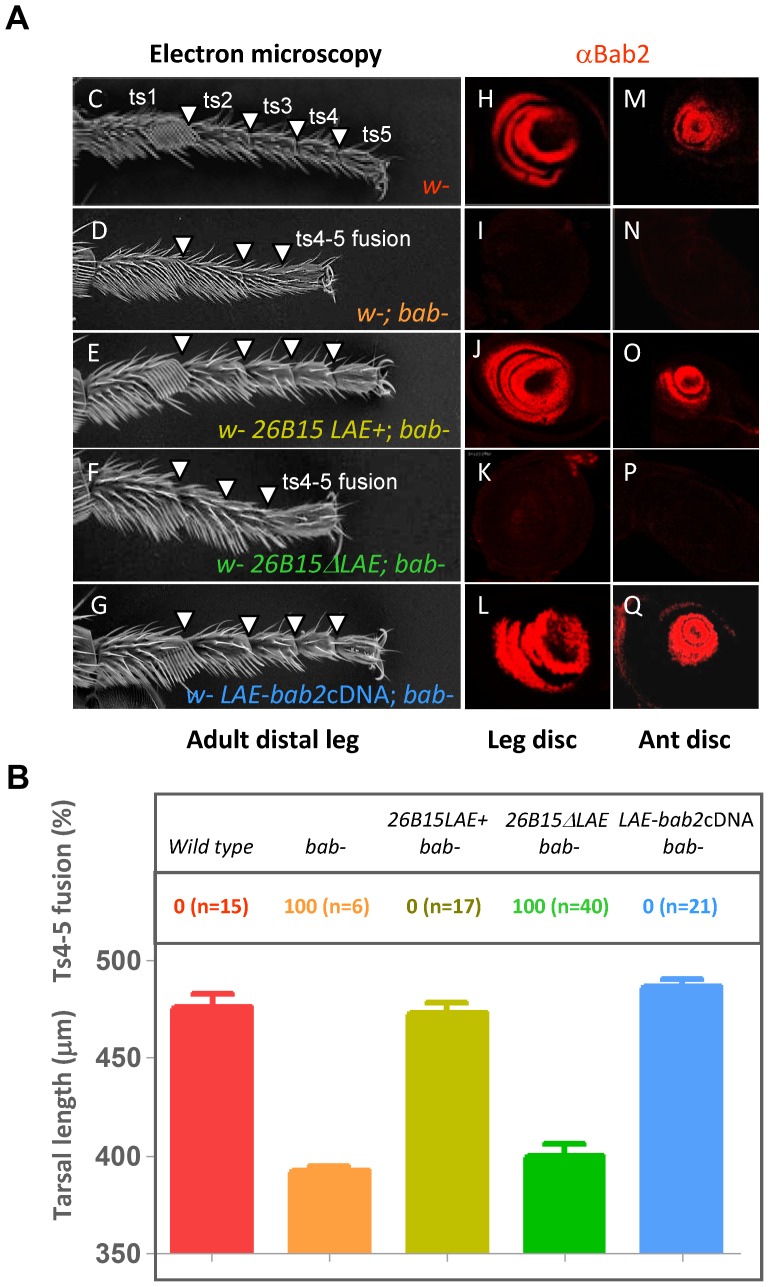
LAE is necessary and sufficient for wild-type *bab2* expression and function *in-vivo*. (A) LAE-containing *bab2*-expressing constructs restore normal leg and expression defects in a loss-of-function context. (C–G) Scanning electron microscope views of distal adult T1 legs from wild-type (C), *bab^AR07^* (D), *w^1118^ 26B15-LAE+*; *bab^AR07^* (E), *w^1118^*; *26B15 ΔLAE*; *bab^AR07^* (F) and *w^1118^ LAE-bab2cDNA*, *bab^AR07^* (G) males, are shown. Wild-type tarsal junctions between ts1–5 are indicated by arrowheads. (H–Q) Bab2 immunostaining (red) of leg (H–L) and antennal (ant) (M–Q) imaginal discs, dissected from wild-type (H and M), *bab^AR07^* (I and N), *w^1118^ 26B15-LAE+*; *bab^AR07^* (J and O), *w^1118^26B15ΔLAE*;*bab^AR07^* (K and P) or *w^1118^ LAE-bab2cDNA*; *bab^AR07^* (L and Q) third-instar larvae, are shown. The null *bab^AR07^* allele [17] corresponds to a large deletion removing the entire *bab1*/*2* intergenic region that contains the LAE, and most of the *bab2* transcription unit (unpublished). Both the *26B15LAE+* PacMan and *LAE-bab2cDNA* constructs rescued the tarsal fusion phenotypes (particularly between ts4–5) and restored the wild-type leg and antennal Bab2-expression patterns. (B) Quantification of mutant phenotypes. A diagram is shown for each genotype with mean and standard error of mean.

To define whether the LAE is also necessary to ensure normal *bab* expression *in-vivo*, we used a *P*[acman] BAC construct (*26B15*) [30], including the *bab2* transcription unit and the LAE-containing intergenic region (see Figure 1A). Adults homozygous for the *bab^AR07^* null allele display shortened tarsi with segmental joint fusions, particularly the fully penetrant fusion of ts4–5 (Figure 2A–B) [18]. A single copy of the intact *26B15* BAC construct was sufficient to restore normal Bab2 protein expression in both developing leg and antenna (Figure 2A, J and O, respectively), as well as to rescue *bab* mutant phenotypes (Figure 2A–B, arrowheads in E), suggesting the lack of a remote shadow enhancer located within *bab1* or elsewhere in the vicinity of the *bab* locus. In contrast, no appendage-specific Bab2 expression could be detected for a LAE-deleted version of the *26B15* BAC construct (Figure 2A–B, K and P). Further, phenotypic rescue could neither be observed (Figure 2A–B, see arrows in F). We conclude that in our experimental conditions the LAE *cis*-regulatory module appears to be strictly required for *bab2* expression and function in developing limbs. Of note, the wild-type *26B15* BAC and its LAE-minus version were both capable of partially restoring dominant abdominal pigmentation defects of *bab^AR07^* heterozygous females (Figure S3) (see discussion).

To ask whether the LAE is also sufficient to ensure normal Bab2 expression in developing limbs, we then tested the ability of an LAE-driven *bab2* cDNA construct (LAE-bab2cDNA) to rescue *bab^AR07^* phenotypes. Normal leg segmentation and limb-specific Bab2 expression were restored by a single copy of the *LAE-bab2cDNA* construct (Figure 2A–B, G, L and Q), demonstrating that the LAE is both essential and sufficient for limb-specific *bab2* expression and function.

### LAE includes shared leg and antennal regulatory information

**Figure 3:**
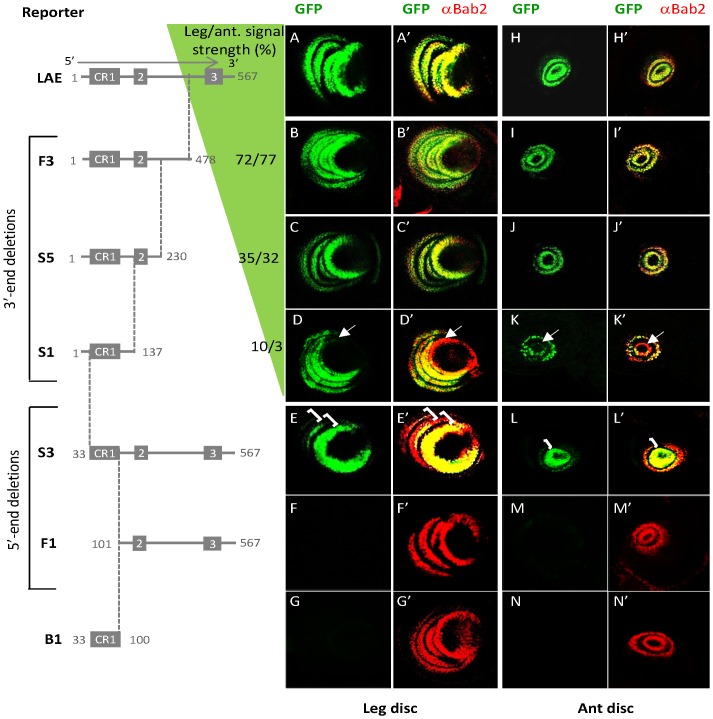
LAE contains separate signal specificity and intensity regions, with shared leg and antennal regulatory elements. The names and relative positions (with respect to the entire LAE) of each genomic fragment tested in our site-directed GFP reporter assay are shown on the left side. The conserved CR1-3 regions are indicated by grey boxes. The relative levels of GFP expression (Materials and methods), in leg and antennal (ant) tissues, are indicated in % relative to the entire LAE. For each examined construct, GFP expression (green) alone and in combination with Bab2 immunostaining (red) of leg (A–G and A′–G′, respectively) and antennal (H–N and H′–N′, respectively) imaginal discs from late third-instar transgenic larvae, are shown on the right side. Both in the leg and antennal tissues, the *S1* (D–D′ and K–K′, respectively) and *S3* (E–E′ and L–L′, respectively) reporter constructs, displayed decreased GFP expression in the distal- or proximal-most *bab2*-expressing rings, respectively, as visualized by the red-orange signals (see arrows and brackets, respectively) instead of yellow colour normally seen in the merged images. Neither the isolated CR1 sequence (*B1* construct) nor the CR2-3 region (*F1*) drove GFP expression in any limb tissue.

To functionally dissect the LAE, we tested serially truncated constructs removing either 3′ or 5′ sequences (Figure 3). Deletion of 89 bp at the 3′-end (*F3* construct), that removed the CR3 motif, led to a modest decrease in GFP expression (72 and 77% of signal strength, compared to the entire LAE, in leg and antennal tissues, respectively) without affecting expression in the right cells (Figure 3, B–B′ and I–I′). Deletion of 248 additional bp (*S5* construct) further reduced the level of expression (35 and 32% in leg vs. antenna) (Figure 3, C–C′ and J–J′, respectively). Deletion of 93 additional bp at the 3′-end (*S1* construct), removing the CR2 sequence, led to a drastic reduction of the signal strength (10 and 3%), with a nearly-complete loss of GFP expression in the distal-most *bab2*-expressing cells, in either leg or antennal tissues (Figure 3, arrows in D–D′ and K–K′, respectively). Of note, a deletion of 37 additional bp, non-strictly-conserved across all Drosophilidae, led to nearly-complete loss of all rings (not shown), indicating their critical role. These 3′-deletion data support the following conclusions: (i) 327 bp from the 3′ half, including CR3, are required for signal intensity; (ii) whereas the remaining 230 bp from the 5′-half, including CR1-2, are sufficient for limb-specific expression, among which (iii) 93 bp encompassing CR2 are required for making the distal *bab2*-expressing ring.

Conversely, a 100-bp 5′ deletion removing the CR1 sequence (*F1* construct) led to complete loss of limb-specific GFP expression (Figure 3, F–F′ and M–M′). This indicates a key regulatory function for CR1 in all *bab2*-expressing leg and antennal cells. Nevertheless, the 68-bp CR1 sequence alone (*B1* construct) did not drive either leg- or antennal expression (Figure 3, G–G′ and N–N′, respectively). These data indicate that CR1 is critical but not sufficient for GFP-reporter activity in both developing appendages. As the CR2-containing *F1* construct deleted for CR1 did not allow GFP expression (above), we conclude that CR2 is also not sufficient for LAE activity in the distal-most *bab2*-expressing ring.

Given that the CR1 sequence is not sufficient for LAE activity, we then examined the functional significance of the poorly-conserved 32-bp-long 5′-flanking region (see Figure S2). Its deletion (*S3* construct) led to reduced expression levels in the two proximal-most leg rings (spanning tarsi ts1–2 and ts2–3) and the proximal antennal ring (spanning a3–4 segments) (Figure 3, brackets in E–E′ and L–L′, respectively), but specificity retained (i.e., expression in the right cells at the late L3 stage).

Taken together these data indicate that (i) the entire LAE is required to recapitulate normal spatial, temporal and quantitative *bab2* expression patterns; (ii) leg and antennal expression employ shared regulatory information; (iii) the 337 bp 3′-part is only required for signal strength; and finally (iv) the 230 bp 5′-part is critical for signal specificity, with a key role of the CR1 sequence, while the 32 bp 5′-end and CR2 sequences are required quantitatively for normal expression levels in the proximal- and distal-most rings, respectively.

### CR1 includes separate activating and repressive regulatory elements

**Figure 4:**
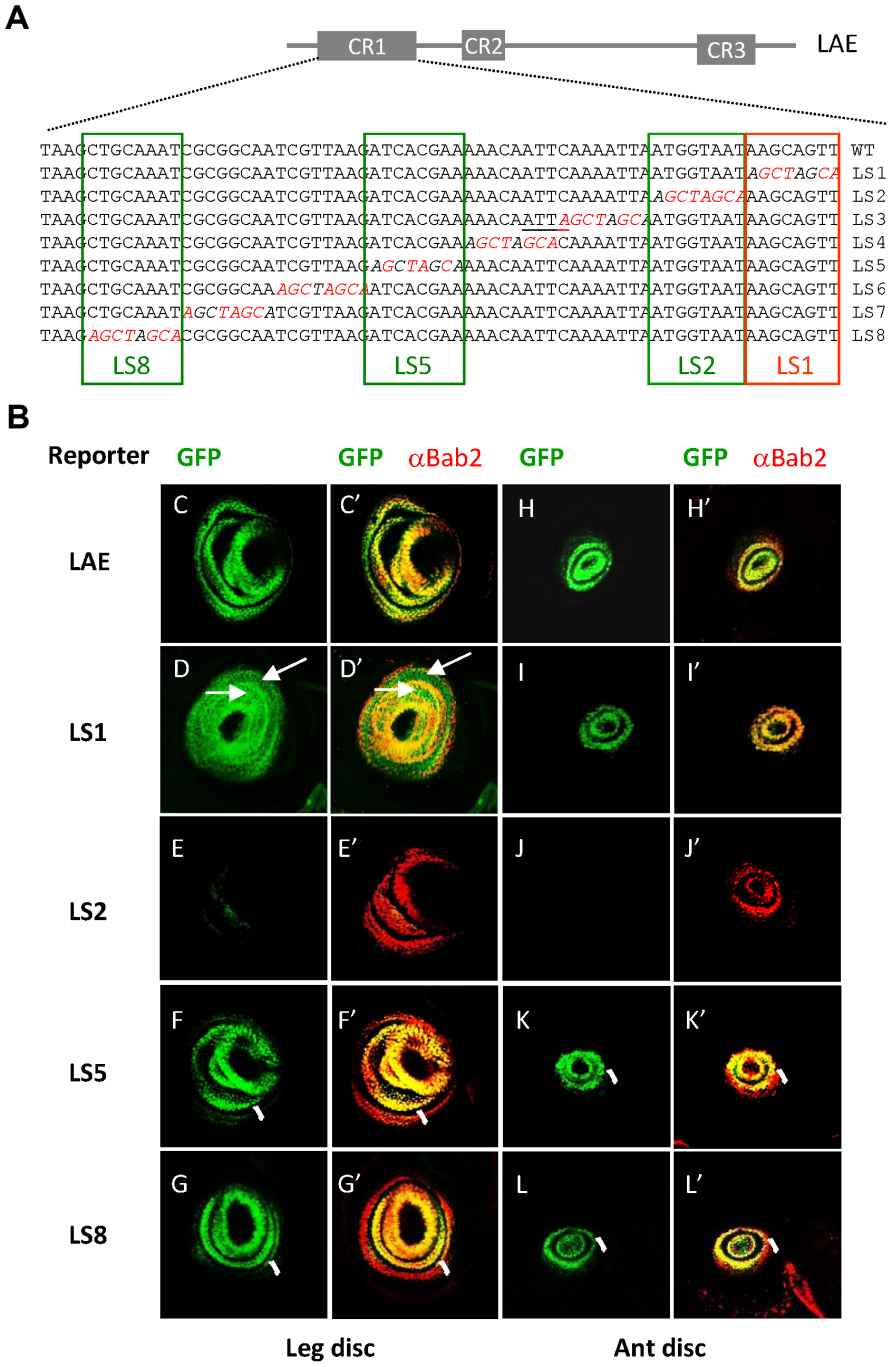
The key CR1 sequence includes separate activating and repressive regulatory information. (A) Linker scanning (LS) mutagenesis of the 68-bp CR1 conserved region. The sequences of the wild-type and of each of the eight mutated (LS1–8) CR1 sub-regions (tested within the entire LAE) are shown, with the names of the examined *LS* constructs indicated on the right side. The inserted linker sequence (AGCTAGCA) is italicized with nucleotide substitutions depicted in red. Note that an additional consensus HD-binding site (underlined) has been created in the LS3 construct. The most striking positive and negative regulatory elements are framed in green and red, respectively. (B) Critical DNA elements within CR1. GFP expression (green) alone and in combination with Bab2 immunostaining (red) of leg (C–G and C′–G′, respectively) and antennal (ant) (H–L and H′–L′, respectively) imaginal discs from late third-instar larvae, expressing either the *LAE-GFP* construct or any of the four LS-mutated derivatives displaying the strongest abnormal expression patterns, are shown (for the remaining four constructs, see Figure S4). *LS2*-driven GFP reporter expression was drastically reduced, in both leg (E–E′) and antennal (J–J′) imaginal discs. Conversely, the *LS1-GFP* construct displayed inter-ring de-repression in leg tissues (D–D′), as determined by the green (arrows) instead of yellow colour normally seen in the merged images. Finally, the *LS8* and to a lesser extent *LS5* constructs exhibited strongly decreased GFP expression in the proximal-most *bab2*-expressing tarsal (F–G) and antennal (K–L) as determined by the red staining (brackets) instead of the yellow colour normally seen on the merge.

Its central role in limb-specific expression led us to dissect the 68-bp-long CR1 sequence by substituting each 8 bp by a linker sequence (see Materials and methods) (Figure 4A). Though all eight CR1-mutated LAE constructs *(LS1-8*) detectably affected GFP expression qualitatively and/or quantitatively (Figures 4B and S4), four (*LS1*, *2*, *5* and *8*) displayed strong defects. *LS2* showed almost complete loss of GFP expression in both leg and antennal tissues (Figure 4B, E–E′ and J–J′, respectively), indicating it affects a crucial positive input. *LS1* led to strong up-regulation in inter-ring cells in the developing leg (Figure 4B, arrows in D–D′), suggesting a distinct role in inter-ring suppression. *LS5* showed globally decreased GFP expression (30%), but more pronounced in the proximal-most leg and antennal ring (Figure 4B, brackets in F–F′ and K–K′, respectively). Finally, *LS8* displayed decreased GFP expression specifically in the proximal-most *bab2*-expressing ring, again in both leg and antennal tissues (Figure 4B, brackets in G–G′ and L–L′, respectively). As to milder effects of other mutants, (i) the *LS4* and *LS6* constructs displayed a partial GFP intensity decrease in the proximal-most ring, particularly marked in leg tissues; (ii) *LS7* showed a partial inter-ring de-repression in leg tissues; (ii) whereas *LS3* and *LS6* exhibited a slight GFP intensity decrease specifically in antennal tissues (50% each) (Figure S4).

Taken together these site-directed mutagenesis data indicate (i) tightly-associated leg and antennal CR1 regulatory information, and (ii) that the LS2 sub-sequence is indispensible for LAE activity.

### Distal-less is the central direct *bab2* activator in developing appendages

**Figure 5:**
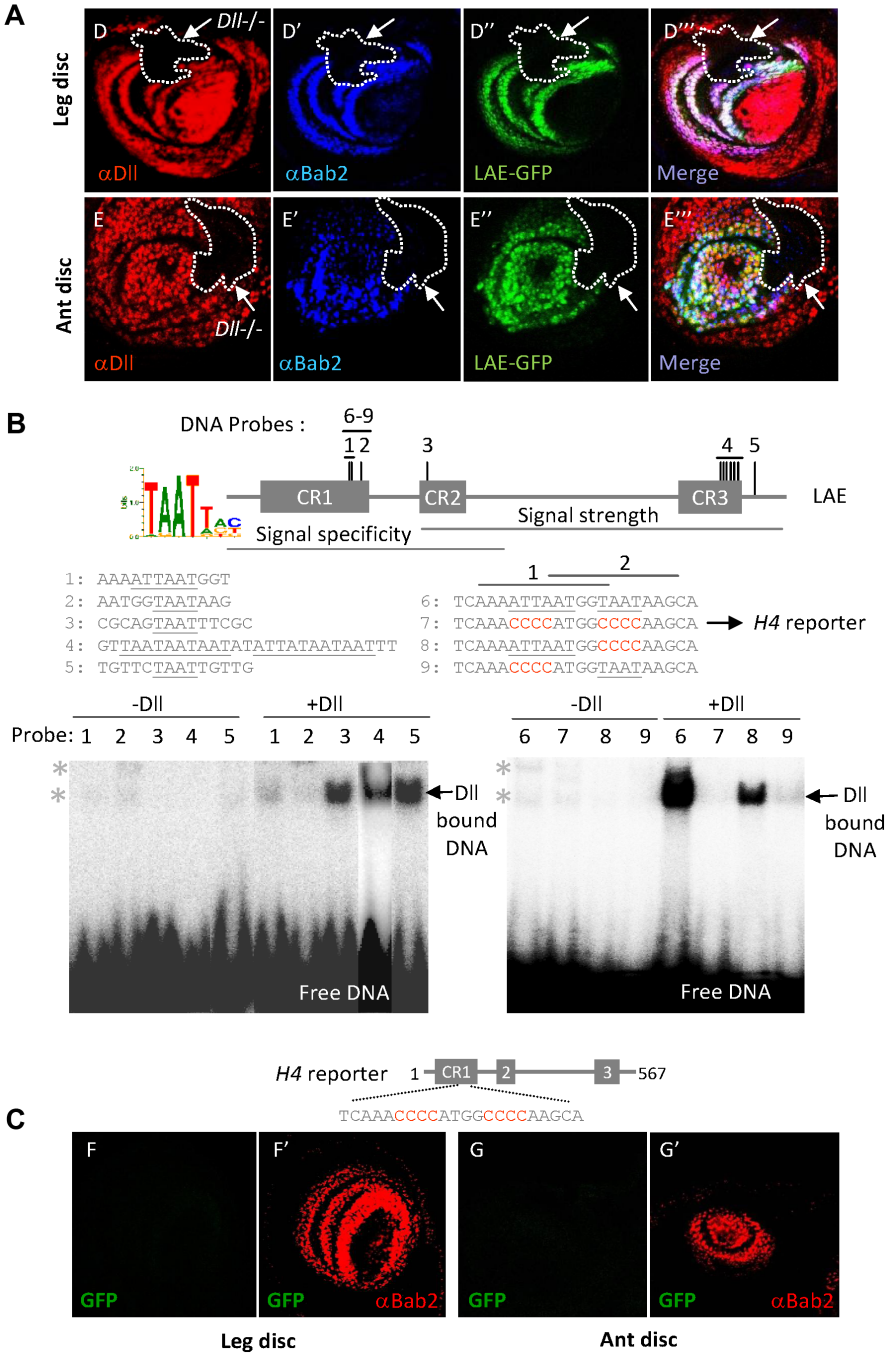
The Dll homeodomain protein positively regulates LAE activity through direct binding to CR1 sequences. (A) *Dll* activity is critically required for both *bab2* and *LAE-GFP* expression in developing limbs. Dll immunostaining (red), Bab2 immunostaining (blue) or GFP expression (green) alone, as well as the corresponding merged images, of leg (D–D″′) and antennal (ant) (E–E″′) imaginal discs from late third-instar larvae carrying *Dll* null mutant clones, are shown. Large *Dll^SA1^* mutant clones (circled by dashed white lines) are indicated by white arrows in all panels. Expression of both endogenous *bab2* and *LAE-GFP* reporter was cell-autonomously extinguished in the *Dll* mutant cells. (B) Dll binds *in-vitro* to HD-binding motifs located throughout the LAE sequence. The extents of the signal specificity and strength regions are indicated. The sequence LOGO of the consensus Dll-binding site (mainly composed of a TAAT motif), as determined from selection in bacteria [52], is shown in the left side. The positions of 11 TAAT/ATTA motifs within the LAE are indicated by vertical lines, and the corresponding DNA probes (#1–9) used in the EMSA experiments indicated above. Of note, all sites but one (probe #5) are situated within the CR1–3 conserved regions. LAE sequences (in grey) included in each labelled double-stranded DNA probe are indicated beneath, with mutated nucleotides shown in red. PhosphorImager detection of DNA-protein complexes separated by electrophoresis on native polyacrylamide gels is shown on the right. *In-vitro* translated Dll was omitted or added, as indicated above the lanes, numbered according to the tested probe. The positions of non- and Dll-specific shifted DNA-protein complexes are indicated by asterisks and a horizontal arrow, respectively. The extended probes #6–9 included the overlapping DNA fragments #1–2. In contrast to the two singly-mutated probes (#8–9), the high-affinity Dll-specific complex detected with the wild-type sequence (#6) was no longer detected with the doubly-mutated derivative (#7). (C) The triple TAAT-containing sequence within CR1 is critical for LAE activity *in-vivo*. GFP expression (green) alone and in combination with Bab2 immunostaining (red) of leg (F–F′) and antennal (G–G′) imaginal discs from a late third-instar larva expressing the *H4-GFP* reporter construct, a *LAE-GFP* derivative mutated on the three CR1 TAAT/ATTA motifs (identical to EMSA probe #7), are shown. No GFP expression could be detected, either in leg or in antennal tissues.

Having identified key *cis*-regulatory DNA elements, we next sought positively acting factors that directly bind there. We noticed that the critical LS2 motif is embedded within an A/T-rich sequence (AAAATTAATGGTAATAA), including three potential homeodomain (HD)-binding sites (TAAT or ATTA motifs) of which two were disrupted in the LS2 mutant. Given that HD-containing Dll protein is cell-autonomously required for *bab2* expression [21], we therefore examined whether the *LAE-GFP* reporter also requires *Dll* activity, using clonal analysis. As for endogenous *bab2*, GFP expression was abolished in mutant leg and antennal clones for *Dll^SA1^*, a protein-null allele (Figure 5A, D–D″′ and E–E″′, respectively). This indicates that Dll is cell-autonomously required for LAE regulatory activity, and therefore suggest a direct binding of the HD-containing Dll transcription factor to the LAE, potentially through the key activating LS2 motif within CR1.

In addition to the 3 present in CR1, the entire LAE comprises 8 additional putative HD-binding sites of which 7 are clustered within the CR2 and CR3 sequences (Figures 5B and S2). To test whether Dll is able to bind *in-vitro* to the TAAT/ATTA-containing LAE sequences, we used an electrophoretic mobility shift assay (EMSA). All LAE fragments including one to six of the 11 TAAT/ATTA motifs bound *in-vitro* translated Dll, albeit with distinct affinities (Figure 5B). Unexpectedly, given the key role *in-vivo* of the LS2 region, each TAAT or ATTA site in CR1 showed rather low *in-vitro* affinity (DNA fragments #1–2), when tested individually. We therefore examined whether Dll binds with a higher efficiently to a larger DNA fragment including the three TAAT/ATTA motifs present in CR1 (i.e., AAAATTAATGGTAATAA). As a matter of fact, Dll strongly bound this extended fragment (#6) (Figure 5B). Furthermore, stable interaction was strictly dependent upon the three TAAT/ATTA motifs. Whereas singly-mutated (TAAT vs. CCCC) fragments bound Dll with lower affinity, the mutation of the double overlapping TAAT/ATTA sites (ATTAAT vs. CCCCAT; probe #9) showed a stronger effect, while Dll binding was abolished when all three TAAT/ATTA motifs were mutated (probe #7).

To investigate whether the extended AT-rich sequence mediates *in-vivo* regulation by Dll, we then introduced the same TAAT-mutated sequence (i.e., CAAACCCCATGGCCCCAAGCA) into the *LAE-GFP* reporter (*H4* construct). The *H4* construct no longer expressed GFP either in leg or antennal tissues (Figure 5C, F–F′ and G–G′, respectively), indicating that these three HD-binding sites are crucial for LAE activity *in-vivo*. Surprisingly, mutation of the two overlapping TAAT/ATTA motifs was silent *in-vivo* (LS3 mutant; Figure 4B). In fact, a new HD-binding site capable of stably interacting with Dll *in-vitro* (not shown) has been fortuitously created in LS3 (see Figure 4A), providing a likely rationale for this discrepancy.

Taken together, *in-vitro* and *in-vivo* data establish that Dll-dependent activation of *bab2* expression in developing leg or antenna, involves direct binding to the conserved HD-binding sites of the CR1 regulatory sequence.

### Rotund only contributes to *bab2* activation in proximal leg and antennal rings

**Figure 6:**
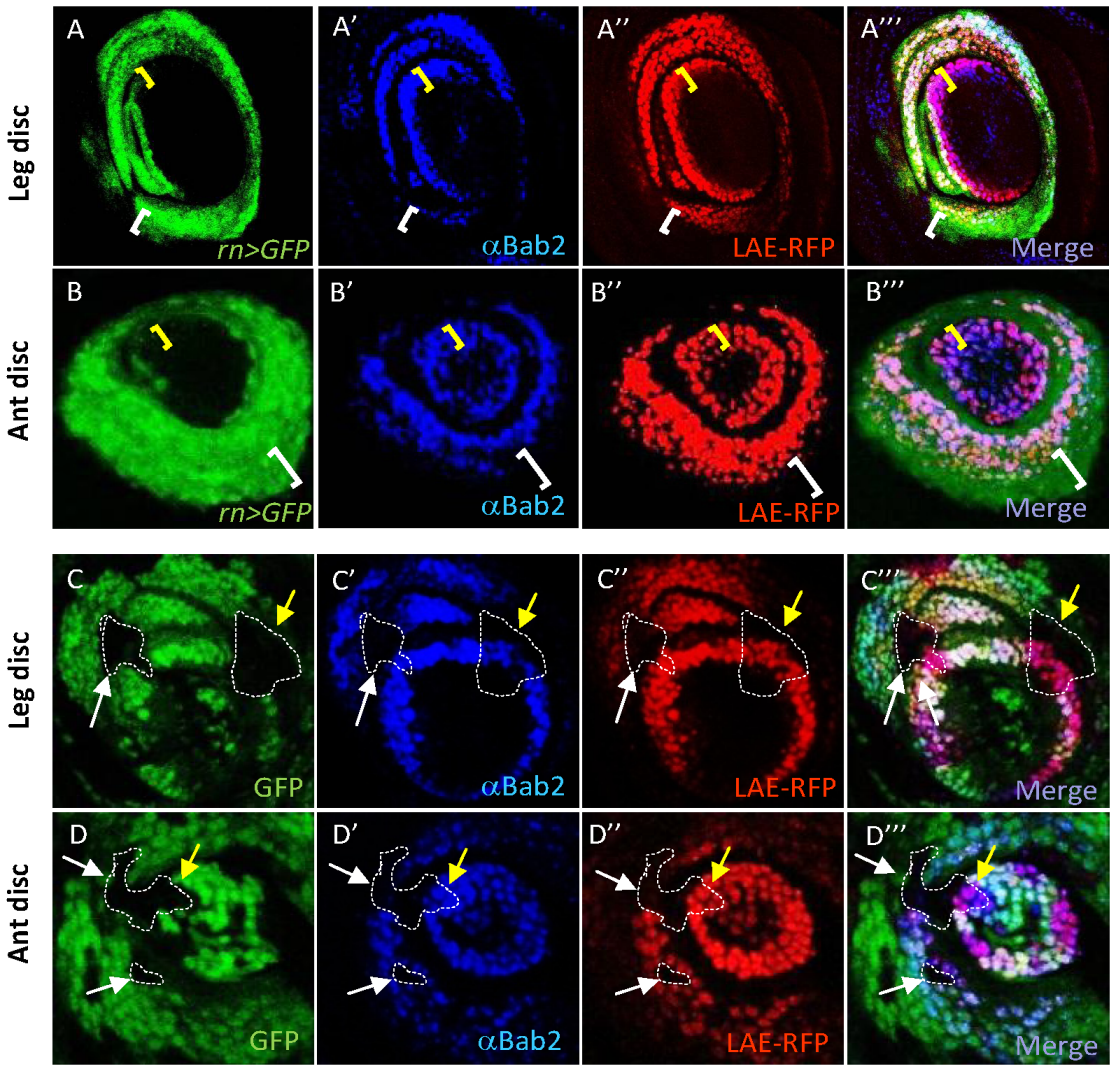
*rotund* is required for *bab2* activation only in proximal-most rings, in both leg and antenna. GFP expression (green), Bab2 immunostaining (blue) and *LAE-RFP* expression (red) alone, as well as the merged images of leg (A–A″′ and C–C″′) and antennal (ant) (B–B″′ and D–D″′) discs from late third-instar larvae, either wild-type (A–B) or carrying *rn* null mutant clones (C–D), are shown. *rn* expression was monitored by combining *rn-Gal4* and *UAS-GFP* constructs (*rn*>GFP). LAE regulatory activity was monitored with a *LAE-RFP* construct (Materials and methods). *rn*-*Gal4* reporter expression is not detected in the distal-most *bab2*-expressing cells (yellow brackets), and extends more proximally than *bab2* expression (white brackets). In mosaic tissues, *rn^16^* mutant clones (examples are circled by dashed lines) are detected by the absence of GFP (black areas). Note that only proximally-located mutant cells (white arrows), and not those located in the distal-most ring (yellow arrows), display strongly diminished *bab2* and *LAE-RFP* expression, in either leg or antennal tissues.

Having shown above that normal LAE activity in the proximal-most *bab2*-expressing cells is mediated by its 32-bp 5′-end region (R32) (Figure 3, E–E′ and L–L′), we then sought for candidate transcription factors encoded by known limb P-D patterning genes. One, *spineless*, has been previously shown to regulate *bab2* expression in the leg proximal-most rings [19]. However, we found no evidence for any putative binding site for the Ss/Tango bHLH-PAS heterodimer (GCGTG) [31], [32] in R32, and even in the entire LAE. A second candidate was *rotund* (*rn*), a *spineless* target gene [9], [33] encoding a C2H2 zinc-finger protein and whose expression corresponds to proximal tarsal segments [9]. First, we compared expression patterns of both genes, using *rn-Gal4* and *UAS-GFP* constructs. GFP expression takes place in the right cells and at the right time [34], but is still detected beyond the mid L3 stage due to perdurance of Gal4 and GFP proteins. At late L3 stage, perduring *rn-Gal4* expression is only detected in the proximal-most Bab2-expressing tissues, and extends more proximally than *bab2*, particularly in antennal tissues (Figure 6, A–A″′ and B–B″′; see yellow and white brackets, for distal versus proximal *bab2*-only and *rn*-only expressing cells, respectively). To analyse the role of *rn* on *bab2* and LAE-driven expression, we generated mitotic clones (GFP deficient) of cells homozygous for the *rn^16^* null allele. Strong cell-autonomous reduction of endogenous *bab2* (in blue) and *LAE-RFP* reporter (in red) expression was then observed for leg and antennal clones overlapping the proximal-most *bab2*-expressing rings (Figure 6, C–C″′ and D–D″′, respectively). This indicates that *rn* activity on *bab2* is spatially restricted and contributes to *bab2* expression only in ts1–2 leg and a3–4 antennal tissues.

### Rotund directly activates *bab2* through the LAE T-rich 5′end region

**Figure 7:**
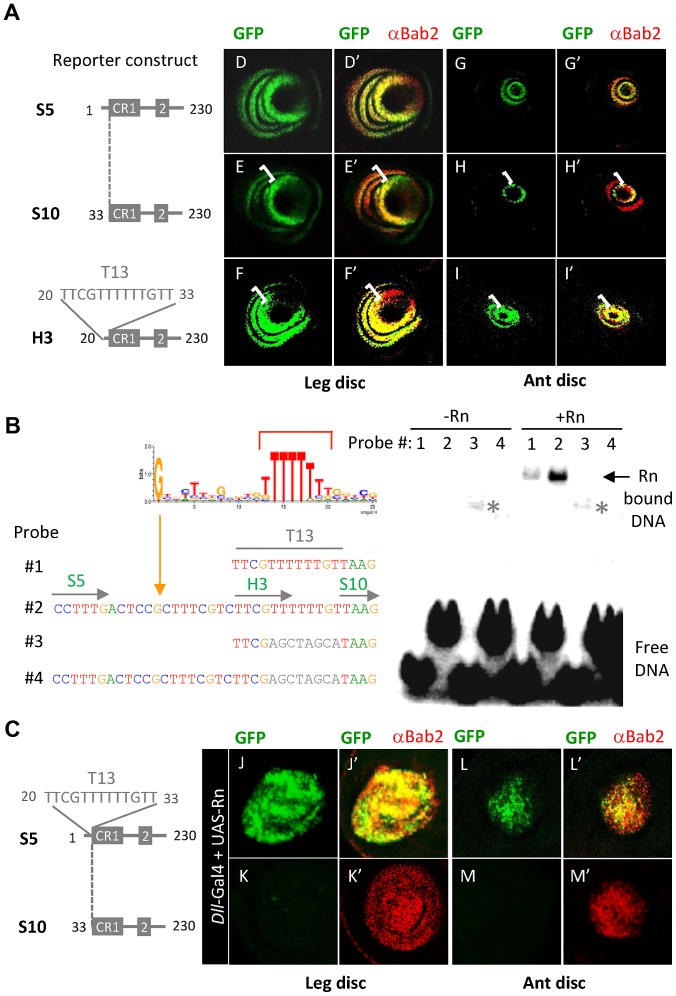
Rotund Zinc-finger protein positively regulates *bab2* expression through direct binding to the T13 LAE sequence. (A) The R32 sequence is required for normal *LAE-GFP* reporter activation in the proximal-most *bab2*-expressing cells. GFP expression (green) alone and in combination with Bab2 immunostaining (red) from late L3 leg (D–F and D′–F′, respectively) and antennal (ant) (G–I and G′–I′, respectively) imaginal discs, expressing a given *LAE-GFP* truncated derivative (left side), are shown. The *S5-GFP* construct drove wild-type leg (D–D′) and antennal (G–G′) *bab2* expression patterns. In contrast, GFP expression in the proximal-most *bab2*-expressing cells of the R32-truncated derivative *S10-GFP* was much more weakly detected in leg (E–E′) and not at all in antennal (H–H′) discs (see brackets). The addition of the R32 subsequence T13 to the *S10-GFP* construct (*H3* derivative) was sufficient to increase proximal GFP expression levels (F–F′ and I–I′, see brackets), particularly in antennal tissues. (B) Rn binds *in-vitro* to R32. The sequence LOGO of the consensus Rn-binding site, including a T-rich track (red bracket), is shown in the upper left side. The LAE sequences included in the four labelled double-stranded DNA probes are indicated beneath, with the same color code. In probes #3–4 the wild-type T6 track was substituted by a linker sequence shown in grey. The position of a consensus G is indicated by a vertical orange line. EMSA experiments are shown on the right. *In-vitro* translated Rn protein was omitted or added as indicated, and line numbers refer to the four tested probes shown on the left. The positions of non- and Rn-specific shifted DNA-protein complexes are indicated by an asterisk and a horizontal arrow, respectively. Note that Rn bound T13 less strongly than the entire R32 DNA fragment. (C) The R32 sequence is critical for *rn*-induced *LAE-GFP* expression in developing limbs. GFP expression (green) alone and in combination with Bab2 immunostaining (red) from late L3 leg (J–K) and antennal (L–M) imaginal discs, over-expressing Rn under the control of the *Dll* regulatory sequences (*Dll*-Gal4 plus *UAS-Rn* constructs), are shown. When Rn was over-expressed throughout the *Dll* domain, endogenous *bab2* and the *S5-GFP* reporter construct were both up-regulated in nearly all Dll-expressing cells, in either developing leg or antenna. In striking contrast, GFP expression in *S10* reporter tissues is not detected, indicating that the R32 sequence is critically required for Rn-mediated *S5-GFP* expression.

Next, we examined whether the Rn protein activates *bab2* expression via the R32 sequence at the LAE 5′end, specifically required for GFP reporter expression in the proximal-most tarsal and antennal *bab2*-expressing ring(s). To further confirm its functional requirement, we deleted the R32 sequence in the context of the 230-bp *S5-GFP* construct (Figure 3), recapitulating leg and antennal *bab2* expression, although with lower level than the full size LAE (Figure 7A, D–D′ and G–G′, respectively). The R32-deleted *S5* fragment (*S10*) drove relatively strong GFP expression in the distal-most ring but only very weakly in the proximal-most ring(s), particularly in antennal tissues (Figure 7A, brackets in E–E′ and H–H′). These data confirm that the R32 sequence is required for full LAE-driven expression in proximal *bab2*-expressing cells in both tarsal and antennal tissues.

The R32 sequence includes a 6-bp-long oligo-T track (T6) embedded within a 13-bp-long T-rich (T13) sequence (TTCGTTTTTTGTT), that resembles binding sites for the vertebrate Rn homolog [35]. To test whether the Rn protein is able to bind effectively to T13 in LAE, EMSA experiments were performed with DNA probes covering either the T13 sequence (probe #1) alone or the complete R32 sequence (#2) (Figure 7B). Though *in-vitro* translated Rn bound both probes, the R32 fragment was bound about 5-fold more than T13 (Figure 7B, compare lanes 1 and 2). R32 includes a sequence matching the consensus binding site for *Drosophila* Rn, as determined by recent bacterial one-hybrid binding site data [32] (see Figure 7B). The T6 track appeared critical for specific binding, as R32 and T13 fragments mutated in the T6 track (probes #3–4) were not stably bound by Rn. The EMSA experiments thus indicate that the LAE T13 region constitutes a strong binding site for Rn.

To examine the functional importance of the Rn binding site *in-vivo*, we added the T13 sequence to the truncated *S10* construct to yield the *H3* construct. We found that the *H3-GFP* reporter activity in leg and antennal tissues was similar to that of *S5-GFP* (Figure 7A, brackets in F–F′ and I–I′, respectively). Even though the GFP expression level remained somewhat low in the developing antenna, we conclude that the T13 sequence is sufficient for GFP-reporter activation in the proximal-most *bab2*-expressing rings in both the leg and antennal imaginal discs. To further confirm that T13 is essential to mediate direct up-regulation of *bab2* by the Rn activator, we performed *rotund* gain-of-function (GOF) experiments, using the *S5* and *S10* reporter constructs. Ectopically-expressed Rn (*Dll*>Rn) activated endogenous *bab2* and *S5-GFP* expression throughout the Dll-expressing leg and antennal cells (Figure 7C, J–J′ and L–L′, respectively), whereas the T13-deficient *S10* construct displayed GFP expression neither in leg nor in antennal tissues (Figure 7C, K–K′ and M–M′, respectively). Surprisingly, the normal GFP-expressing rings of cells were also no longer detected, suggesting that Rn protein behaves as an indirect repressor, in addition to being a direct *bab2* activator through the T13 sequence.

Taken together with the clonal analyses, we deduce that (i) Rn activity is necessary (in proximal but not in distal rings) and sufficient (when ectopically expressed throughout the Dll domain) for *bab2* and LAE-driven activation and (ii) this regulation is direct, via Rn binding to the LAE T13 sequence.

### Joint ectopic expression of Dll and Rn is sufficient to activate *bab2* in dorsal limb tissues

The above experiments indicated that both Dll and Rn are required together for full *bab2* and LAE-dependent reporter activation. This raised the possibility that Dll and Rn together can instruct cells to up-regulate the LAE. To investigate their joint instructive properties, we mis-expressed Dll, Rn or Dll+Rn in wing, haltere and eye tissues using flip-out GOF experiments [36]. *bab2* is weakly expressed in restricted domains in developing dorsal appendages (around the wing pouch and in the haltere pouch [19]) but silent in eye tissues. Of note, LAE-driven reporter expression could not be detected either in developing wing, haltere or eye (not shown).

**Figure 8:**
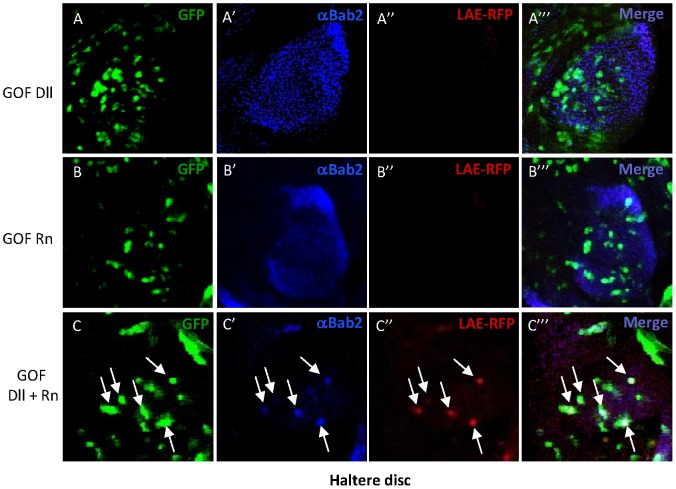
Joint over-expression of Dll and Rn is sufficient to cell-autonomously activate *bab2* in haltere cells. Joint over-expression of Dll and Rn proteins is sufficient to cell-autonomously activate *bab2* in developing haltere pouch tissues. GFP expression (green), Bab2 immunostaining (blue) and RFP expression (red) alone, as well as the merged images, of the pouch tissues from haltere imaginal discs of *LAE-RFP* third-instar larvae carrying flip-out clones over-expressing either Dll (A–A′″), Rn (B–B″′) and Dll plus Rn (C–C″′), are shown. Flip-out clones (GFP+) were induced by a single heat shock in second or third instar larvae (see Materials and methods). Contrary to mosaic larvae carrying haltere pouch cells expressing either Dll or Rn alone, respectively, most of the clones (some are indicated by arrows) over-expressing both Dll and Rn cell-autonomously up-regulate *bab2* and *LAE-RFP* expression, indicating a functional synergism between these transcription factors.

Sustained ectopic expression of Dll protein in developing wing disc induces the entire leg P-D differentiation program including *bab2* expression [37]. We therefore used an *hsp70-Flp* construct to generate small or even single cell clones through heat induction in second- or third-instar larval tissues. In such conditions, mis-expressed Dll appeared inefficient in ectopically activating *bab2* as well as *LAE-RFP* expression in eye, wing and haltere discs (0/50, 9/180 and 0/150 examined clones, respectively) (Figure 8, A–A″′; not shown). Significantly, the few RFP positive clones in the wing disc all corresponded to cells that normally express *bab2* (not shown). In equivalent analyses with mis-expressed Rn, eye, wing and haltere clones induced neither endogenous *bab2* nor *LAE-RFP* expression (n>50 for each tissue) (Figure 8, B–B″′; not shown), even in wing and haltere cells normally expressing *bab2*. In striking contrast, on co-expressing Dll+Rn proteins, a large proportion of examined wing and haltere clones (120/180 and 130/180, respectively) cell-autonomously activated both *bab2* and *LAE-RFP* expression (Figure 8, C–C″′; not shown). Further, in most of the *LAE-RFP* non-expressing wing and haltere clones, Dll protein was not detectably accumulated (not shown). Significantly, eye clones co-expressing Dll+Rn failed to activate endogenous *bab2* and LAE-driven reporter genes (not shown), indicating tissue specificity for their joint instructive properties. These data establish that ectopic co-expression of Dll and Rn is sufficient in instructing dorsal appendage cells to activate endogenous *bab2* and LAE-driven reporter expression, presumably adopting a “proximal-ring transcriptional mode”, supporting (i) their direct binding to the LAE and (ii) a tissue-specific functional synergy involving these two transcription factors.

## Discussion

In this report, we have characterized a 567-bp-long evolutionarily-conserved enhancer element ensuring *bab2* expression in distal leg and antennal epithelial cells, from larval to adult stages. In contrast to the view that enhancer redundancy be a rule of thumb for developmental genes [38], our rescue experiments indicate that this single CRM isolated from 150 kb of the *bric-a-brac* locus is both necessary and sufficient to reliably govern gene expression in distinct limb morphogenetic fields. Further, we have shown that (i) the LAE regulatory activity is under the direct control of Dll, (ii) full expression in proximal-most rings depends on the direct binding of Rn, and (iii) each transcription factor interacts with a single critical binding site. Lastly, joint mis-expression of both Dll and Rn is sufficient to ectopically up-regulate endogenous *bab2* as well as *LAE-RFP* expression in wing and haltere cells.

### Leg versus antennal *bab2* regulation

The *Drosophila* leg and antenna are thought to be homologous structures evolved from a common ancestral appendage, as shown by leg-to-antenna or antenna-to-leg transformations caused by mis-expression of P-D patterning or homeotic genes [16], [22], [39]–[42]. To our knowledge, *bab2* is the first example of a developmental gene for which a single transcriptional enhancer is shown to be both necessary and sufficient to accurately ensure a complex gene expression pattern in distinct limb morphogenetic fields. As none of our mutated LAE constructs specifically affected antennal or leg expression, our findings thus support the idea that an ancestral P-D genetic cascade emerged before limb diversification in insects.

### LAE comprises separate signal specificity and intensity booster regions

*bab2* expression in developing leg and antennal discs is dynamic and complex, going from broad regional expression at early L3 stage to precisely positioned rings, and later on, to graded expression at pupal and adult stages [17]–[19]. Limb-specific regulation occurs through the 230-bp-long 5′ half of the LAE, which integrates positive inputs from both Dll and Rn transcription factors, whereas the 327-bp-long 3′ half appears to be required for signal amplification to confer robust expression, but is unable alone to drive any GFP-reporter expression. The presence of this signal intensity “booster” could explain the apparent lack of shadow enhancer [38], as indicated by our phenotypic rescue experiments (Figure 2). Conversely, the *26B15* BAC construct partially rescues the *bab* mutant abdominal pigmentation defects (Figure S3), suggesting it may contain a shadow enhancer to assist the abdominal *cis*-regulatory elements identified previously within the *bab1* transcription unit (i.e., excluded from the 26B15 BAC; see Figure S3) [27]. Even though we cannot formally exclude that the *bab* locus includes a remote secondary enhancer assisting the LAE in other environmental or genetic backgrounds, our data suggest that a single locus can harbor both partially-redundant (abdominal) and “master” (limb) tissue-specific transcriptional enhancers with distinct functional constraints.

### The Dll protein is a necessary, but not sufficient, general limb-specific *bab2* activator

**Figure 9:**
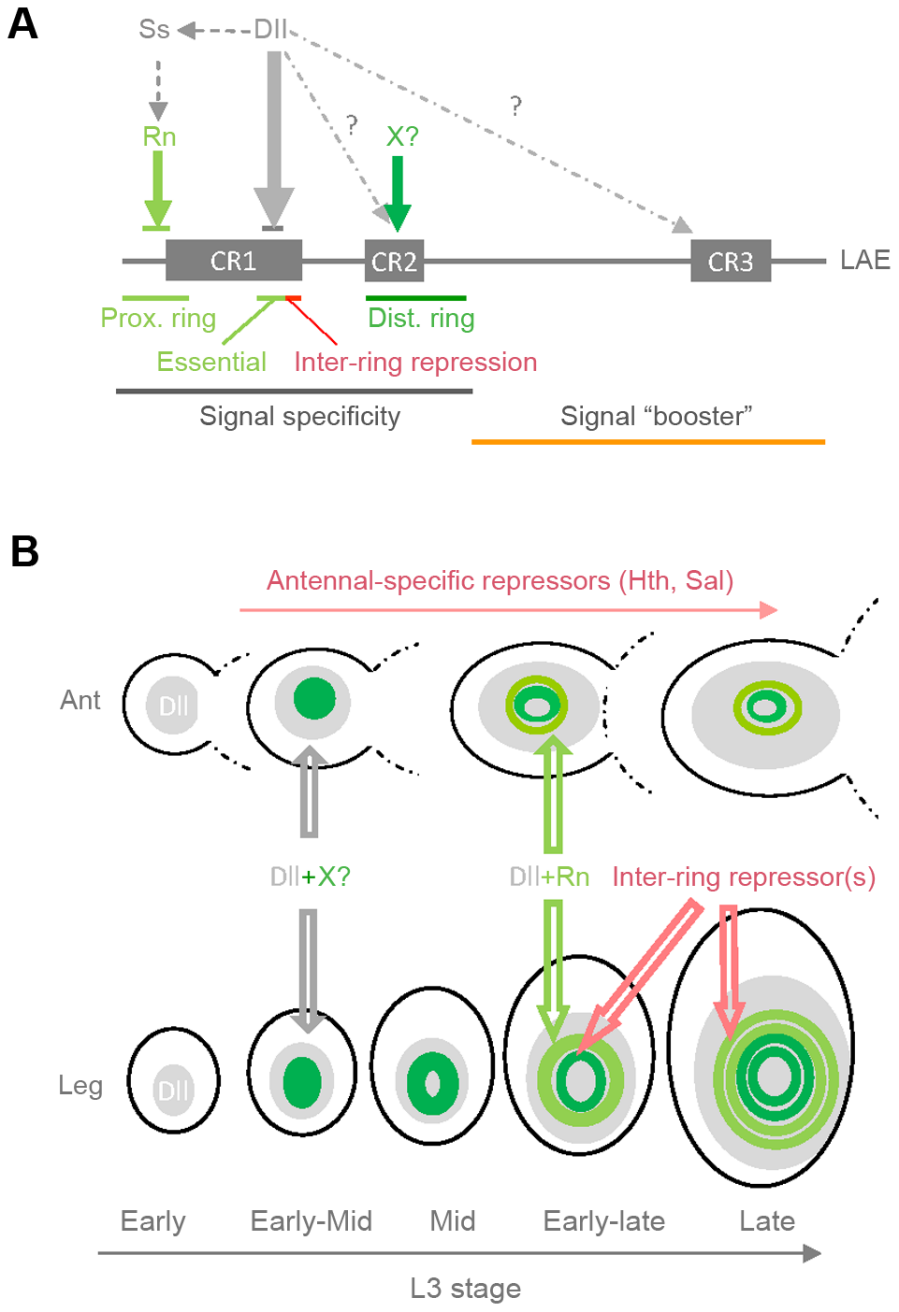
A model for spatio-temporal *bab2* regulation during distal limb development. (A) LAE functional organization. Relevant *cis*-regulatory features are delineated beneath the DNA stretch. Within the 5′-half, critical for distal-limb specificity, key regions required for ring activation and inter-ring repression are shown as green and red lines, respectively. The 3′-half, which is only required for high level of expression (signal amplifier or “booster”), is depicted as an orange line. The binding sites for the direct *bab2* regulators Dll and Rn (in grey and light green, respectively) as well as for an unknown factor (X, in dark green) required for normal distal-most ring expression level, are indicated by vertical arrows. (B) A model for spatio-temporal regulation of *bab2* gene during third-instar larval (L3) stage in antennal (ant) and leg imaginal discs (see main text for details).

In this study, we have established that *bab2* is a direct target of the Dll homeodomain-containing transcription factor, acting through at least the critical AAATTAATGGTAAT composite binding site present in the CR1 sequence (Figure 9A). Interestingly, similar A/T-rich Dll binding sites are also present in enhancers of *ss* and *dac* (i.e., AATTTAATGGTAAA and AAATTATATTTAAT, respectively), two other direct Dll target genes [15], [23], suggesting a conserved CRM grammar for Dll-regulated genes. Although Dll protein is expressed throughout the larval, pupal and adult stages, onset of *bab2* expression starts only at the early-mid L3 stage, in the form of a circular domain within the *Dll*-expressing distal territory (Figure 9B) [19]. Consistently, we have shown that the Dll transcription factor is required but not sufficient for cell-autonomous *bab2* expression (Figure 8A). In addition to the critical CR1 TAAT-rich sequence, we have shown that Dll protein also binds strongly *in-vitro* to the other HD-binding sites (Figure 5B). It is thus formally possible that the Dll transcriptional activator may contribute to the signal intensity “booster” effect of the LAE 3′-half (Figure 9A). However, signal boosting sequences situated in the middle of the LAE (i.e., between positions 231–478; see Figure 3) do not contain TAAT motifs, indicating that at least one other activating transcription factor is involved. In conclusion, in addition to Dll, characterization of new TF(s) interacting with LAE 3′-half sequences may help to better understand CRM activity, both in terms of tissue specificity and expression enhancement.

### Rotund protein: a proximal-specific intermediate between *spineless* and *bab2*?

The specific requirement for *rotund* activity in *bab2* proximal regulation contrasts with data reported in St-Pierre et al. [34], showing apparently normal *bab2* expression in *rn* mutant leg discs. However, although a large *bab2*-expressing ring does indeed appear in mid L3 *rn* mutant larvae, the mature 4-ring pattern never emerges later on, and instead two presumably-distal rings are detected at late L3 stage (not shown). For the first time, we report that ectopically-expressed Rn protein is sufficient to activate and maintain *bab2* throughout the *Dll* expression domain, in both developing leg and antenna (Figure 7C). In light of the dependence of *rn* expression in leg ts1–3 tissues on *ss* activity [9], our data may provide a rationale for why *ss* activity is required in proximal *bab2* regulation [19]. Moreover, Dll and Rn transcriptional activators, that are both necessary for full *bab2* expression in leg and antennal proximal-most ring cells, are also jointly sufficient to ectopically activate *bab2* (and *LAE-RFP* reporter) expression in wing and haltere but not in eye tissues, thus forming a context-specific instructive couple. The molecular basis of their functional synergy remains to be deciphered. Furthermore, in the proximal *bab2*-expressing domain, Dll and Rn are likely to function together with additional, still-unknown activators binding to LS4–5 and LS8 sequences (Figures 4B and S4B), and whose identification will certainly provide insights into the tissue specificity of the LAE.

### Molecular bases for transient activity of Rn on *bab2* expression in developing leg?

Rotund transcription factor is required for proximal *bab2* expression in both legs and antennae. However, unlike antenna, *rn* (and *spineless*) expression is transient in leg tissues. In addition to their previously described roles in ts1–3 growth [9], [33], we propose that transiently-expressed *ss* and *rn* counteract repressive activities of *dac* and/or *bowl*, both of which are dynamically expressed during the critical L3 stage [9], [25]. In fact, de Celis and Bray [25] anticipated that a transiently-expressed *bab2* activator should be present to relieve transient repression by *bowl*. Consistent with this view, *ss* activity represses *bowl* (and *dac*) expression [25], [33], in addition to its role in *rn* activation. As *bowl* (and *dac*) expression has decayed in the tarsal cells which earlier transiently expressed *ss* and *rn*, maintenance of *bab2* expression would no longer require Rn activity. Maintenance of antennal Rn (and Ss) expression may in fact counteract additional *bab2* repressors whose expression persists throughout development, such as *hth* and *spalt* (Figure 9B) [19]. Identification of repressive elements within the LAE will help address these issues. None of 16 different LAE reporter constructs displayed detectable de-repression in the proximal- and distal-most territories, suggesting thus the existence of functionally-redundant repressive DNA elements or alternatively of a competition between transcriptional repressors and activators for binding to the same sites. Of note, site-directed mutagenesis identified a leg inter-ring repression element (LS1 motif) at the CR1 3′-end. Inter-ring repression has been linked to Notch signalling [25]. However, the absence of putative Su(H) binding sites from the LAE sequence suggests that Notch-mediated repression is likely to be indirect. As the LS1 repressive motif is precisely located in the immediate vicinity of the critical composite HD-binding site (Figure 9A), competitive binding processes may well operate between Dll and LS1-bound repressor(s). Whatever the nature of the latter, a functional link with Dac, a non-specific DNA binding protein known to function as part of a multi-protein complex [43], certainly will deserve to be investigated.

### A model for *bab2* regulation in developing limbs

Our findings, coupled with results of previous studies [19], allow us to propose a model for distal limb-specific *bab2* regulation (Figure 9). *bab2* expression starts during the early-mid L3 stage at about 84 hours (hr) after egg laying (AEL) as a circular domain nested within the earlier-initiated *Dll* expression domain [44]–[46]. Our data indicate that Dll is required but not sufficient to activate *bab2*. We suppose that a hypothetical distal activator (X) binding to CR2 (Figure 9A) may contribute to the onset of *bab2* expression. In response to EGFR signalling, *bab2* down-regulation in the distal-most leg territory occurs in mid L3 tissues (at ∼90–96 hr AEL), giving rise to a single large ring (Figure 9B, depicted in dark green). Note that EGFR-mediated *bab2* repression has not been yet described in the distal antenna. From about the same stage, as *rotund* expression starts (∼84–96 hr AEL), a second ring emerges proximally, both in the antenna and the leg tissues (Figure 9B, light green). By late L3 stage (120 hr AEL), *bab2* expression consists of two well-separated rings in the developing antenna and of four concentric rings of distinct intensities in the developing leg. The two proximal-most rings (light green), depending on transient Rn activity, and the two distal-most rings (dark green) emerge both by tarsal growth and inter-ring down-regulation (through at least the LS1-binding repressor). Of note, *rotund* activity is indirectly required for the appearance of the two distal-most rings, due to its role in ts3 growth [9]. Lastly, in the developing leg, precise ring positioning depends on repression by EGFR and Notch signalling [21], [24], the molecular bases for their action remaining to be deciphered.

### LAE is under strong evolutionary constraints among Drosophilidae and beyond

The LAE identified in the present work is systematically located between *bab1* and *bab2* transcription units of 22 Drosophilidae species for which the entire *bab* locus sequence is available (not shown). This suggests strong topological constraints during evolution. As (i) the duplicated *bab* genes are merely co-expressed during leg and antennal development [17], (ii) we have shown a critical role of the LAE in ensuring *bab2* expression (this study) and (iii) no other limb-specific *cis*-regulatory regions could be identified within the 150-kb *bab* locus [27], we thus infer that the LAE is likely to reliably govern *bab1* expression as well. Consistent with our assumption that strong functional constraints have operated during evolution, a LAE-like sequence with partially conserved CR1-2 sequences is even present between paralogous *bab1* and *bab2* genes of the tsetse fly *Glossina morsitans* (Figure S5A and not shown), which diverged from Drosophilidae about 260 million years ago [47]. Furthermore, we have shown that the LAE from *D. virilis* ensures normal regulatory functions in *D. melanogaster*. Although strongly conserved among Sophophora subgenus species (i.e. *D. melanogaster* group), the T13 Rn-binding site is poorly conserved in *D. virilis* and related Drosophila subgenus species (Figure S5B). It is formally possible that Rn has been co-opted recently in the Sophophora subgenus. Alternatively, *D. virilis* Rn could act through subgenus-specific LAE sequences. Consistent with this hypothesis, a T13-related sequence, only conserved among Drosophila subgenus species, is located between CR1-2 (Figure S5B). Investigating the molecular basis of Rn action in the positive regulation of *bab2* may provide an entry point to tackle these evolutionary issues.

## Materials and Methods

### Fly stocks, culture and genetic manipulations

*Drosophila* lines were grown on standard yeast extract-sucrose medium. The *vasa*-PhiC31 ZH2A *attP* stock was kindly provided by F. Karch and was used to generate most of the transgenic GFP and RFP reporters and the two *P[acMan]* BAC constructs. *rn* or *Dll* mutant clones were generated by 30 minute (mn) heat shocks at 38°C, in early first- to late second-instar larvae of genotypes: (i) *y w hsFlp*; *FRT82B Ub-GFP*/*FRT82B rn^16^* and (ii) *y w hsFlp*; *arm-lacZ FRT42D/Dll^SA1^ FRT42D*, respectively. Flip-out clones over-expressing either Dll, Rn or both Dll plus Rn were generated by 40mn heat shocks at 38°C, in second- or third-instar larvae of genotypes: (i) *y w LAE-RFP hsFlp*; *Pact>y+>Gal4, UAS-GFP*/*UAS-Dll*, (ii) *y w LAE-RFP hsFlp*; *Pact>y+>Gal4, UAS-GFP*/*UAS-Rn^1^* and (iii) *y w LAE-RFP hsFlp*; *Pact>y+>Gal4, UAS-GFP*/*UAS-Dll UAS-Rn^1^*, respectively. *UAS-GFP.[S65T]*, *UAS-Rn^1^ and rn-Gal4* lines were obtained from the Bloomington stock center. *UAS-Dll* and *Dll^EM212^-Gal4* line were provided by S. Cohen and M. Suzanne, respectively.

### Reporter constructs, mutagenesis, BAC recombineering and germline transformation

Genomic DNA fragments from the *D. melanogaster* or *D. virilis bab* locus were amplified by standard PCR (using the following primer pairs: cccgaattcGCGCCTAACTAGCCAACAAT/cccggatCCTTTGACTCCGCTTTCGTCTTC and cccgaattcGAAACATCACGTTATCTAGCCACA/cccggatccAGAGTTGCTTGCACACACTCAC, respectively; *Bam*HI and *Eco*RI restriction sites are underlined), cloned into pBP-S3aG, a home-made derivative of the *attB*-containing pS3aG plasmid obtained from T.M. Williams and S. Carroll [27]. Of note, the pBP-S3aG vector includes the TATA-less *bab2* minimal promoter. The pLAE-RFP and pLAE-Bab2 plasmid constructs were made by substituting the GFP insert of the pLAE-GFP construct by a pH2B-RFP insert (obtained from A. Vincent) or a *bab2* full-length cDNA (from pNB-bab2), respectively. Site-directed mutagenesis (including linker scanning) was performed by PCR, using the overlap extension method [48]. All constructs were sequence-verified. BAC recombineering and PhiC31-mediated germline transformation were performed as described [49]. The LAE was validated through insertions within several *attP* genomic sites, including the 2A platform on the X chromosome that was used for all constructs reported in this study.

### LAE sequence identification and alignment

The genomic sequences homologous to the *D. melanogaster* LAE were recovered by Blat analysis at the UCSC Genome Browser website (http://genome.ucsc.edu/cgi-bin/hgBlat?command=start) and aligned with MAFFT (http://mafft.cbrc.jp/alignment/server/). The multiple alignment was then shaded with Boxshade (http://www.ch.embnet.org/software/BOX_form.html).

### Immunofluorescence and image quantification

Third-instar larval imaginal discs were prepared and stained using standard procedures. Confocal analyses were done with a LEICA TCS SP5 microscope. Rat anti-Bab2 [17] and rabbit anti-Dll [50] antibodies were used at 1/2000 and 1/200, respectively. For each reporter construct, GFP fluorescence quantification was obtained from 10 distinct T2 leg and eye-antennal discs (i) dissected from late third-instar larvae grown in the same environmental conditions (temperature and larval density), and then (ii) fixed in the same conditions. Confocal images were analyzed with ImageJ software, using the same area (centered on the distalmost ring of cells), laser excitation settings (75% maximal detection) and brightness/contrast image acquisition.

### In-vitro translation and electrophoretic mobility-shift assay

Dll and Rn proteins were synthesized by coupled *in-vitro* transcription/translation with T7 RNA polymerase and rabbit reticulocyte lysate (TNT assay, Promega). pET-Dll and pCS2-MycRn plasmid constructs were obtained from S. Cohen and P. Couso, respectively. EMSA experiments were performed mainly as described [51], using Novex 6% DNA retardation gels (InvitroGene). Probes were assembled from synthetic oligonucleotides including 4 additional G bases at their 5′-ends, labeled with the Klenow fragment of *E. coli* DNA polymerase I in presence of [α-^32^P]dCTP, and purified on mini Quick spin columns (Roche). Specific activities were roughly similar for all tested probes. Free and shifted probes were revealed with a PhosphorImager.

## Supporting Information

Figure S1 GFP-reporter constructs recapitulating leg and antennal *bab2* expression. Leg (A–F) and antennal (ant) (G–L) imaginal discs from late third-instar larvae expressing GFP-reporter constructs (depicted in the left side) shown in Figure 1. GFP expression (green) alone and combined with Bab2 immunostaining (red) are shown for each construct (A–L and A′–L′, respectively). The reporter constructs encompassing the 1.5 kb-long BP47/42 overlapping region, including the 1.5 kb genomic fragment in isolation (construct #7), all faithfully drove limb-specific *bab2* expression.
(TIF)

Figure S2 LAE sequence alignment from 22 *Drosophila* species. The genomic sequences homologous to the *D. melanogaster* LAE were recovered by Blat analysis at the UCSC Genome Browser website (http://genome.ucsc.edu/cgi-bin/hgBlat?command=start) and aligned with MAFFT (http://mafft.cbrc.jp/alignment/server/). The multiple alignment was then shaded with Boxshade (http://www.ch.embnet.org/software/BOX_form.html). The large (>20 bp) highly-conserved regions (CR1–3) are framed. Notice that *D. mojavensis* and *D. willistoni* LAE sequences have long inserts (1–2 kb) between the CR2 and CR3 regions.
(PDF)

Figure S3 The 26B15 BAC partially rescues *bab* mutant abdominal phenotypes, independently of the LAE. (A) The 150-kb *bab* locus is shown (see Figure 1A). The position of the *bab2*-containing 26B15 BAC (in blue) used for phenotypic rescue experiments is indicated, with the internal LAE depicted as a red box. The positions of the two abdominal-specific *cis*-regulatory elements, within the *bab1* transcription unit, are indicated as yellow boxes. (B) Dorsal views of female abdomens from the wild-type (C) and *bab^AR07^* heterozygous, carrying none (D) or a *26B15 BAC* construct copy either unmodified (E) or LAE-deleted (F), are shown. Whereas pigmentation of wild-type abdominal tergites on segment A2–6 is limited to posterior stripes, females carrying a single *bab^AR07^* allele display nearly fully-pigmented A5–6 segments, evoking male-specific pigmentation. For the A5–6 segments of each genotype, pigmentation extends toward the anterior are indicated by solid bars. The *bab* mutant pigmentation defects are partially rescued in females carrying either an unmodified or a LAE-deleted 26B15 BAC construct.
(TIF)

Figure S4 A linker-scanning mutagenesis of the critical CR1 region reveals functionally relevant motifs. (A) The sequences of the wild-type and of each mutated CR1 sub-region are shown in beneath the entire LAE structural organization, as determined by evolutionary conservation among Drosophilidae (Figure S2). For each of the eight mutated constructs, the inserted linker sequence (AGCTAGCA) is italicized with nucleotide substitutions depicted in red. Positively- or negatively-acting elements are framed in green or red, respectively, dashed lines indicating partially redundant functions. (B) Leg (C–G) and antennal (ant) (H–L) imaginal discs from late third-instar larvae, expressing either the unmodified *LAE-GFP* construct or one out of its four LS-mutated derivatives not shown in Figure 4. GFP fluorescence emission (green) in isolation and in combination with Bab2 immunostaining (red) (C–L and C′–L′, respectively), are shown.
(TIF)

Figure S5 LAE sequences have been conserved among Dipterans. (A) Sequence conservation of the limb-specificity LAE portion among Drosophilidae and in the Glossinidae *Glossina morsitans*. *G. morsitans* LAE-like sequence was identified though blast analyses using the Trace archive nucleotide blast server at NCBI (http://blast.ncbi.nlm.nih.gov/Blast.cgi) and aligned with LAE sequences from representative Drosophilidae species, using MAFFT (http://mafft.cbrc.jp/alignment/server/index.html). Homology shading was made using BoxShade (http://www.ch.embnet.org/software/BOX_form.html). The CR1-2 sequences are framed and locations of the Rn and Dll binding sites are indicated above the alignment. Note that the Rn binding site is poorly conserved. (B) Sequence conservation of the CR1-encompassing LAE portion among Sophophora and Drosophila subgenera. Sequences were aligned and processed as above. The structural organization scheme of the entire *D. melanogaster* (Sophophora subgenus) and *D. virilis* (Drosophila subgenus) LAE sequences are shown in the middle part, with the aligned portions depicted with broken lines. The T13 Rn-binding site of *D. melanogaster* and the T13-like sequence of *D. virilis* are well conserved among Sophophora and Drosophila subgenus species, respectively (T-rich sequences are underlined). Note that the T13-like *D. virilis* sequence is located (i) in a 3′-end extended CR1 and (ii) in an inverted orientation.
(TIF)
