# Supplementary figures and images for: Drosophila Distal-less and Rotund Bind a Single Enhancer Ensuring Reliable and Robust bric-a-brac2 Expression in Distinct Limb Morphogenetic Fields
Source: PLoS Genet. 2013 Jun 27;9(6):e1003581. doi: 10.1371/journal.pgen.1003581 (PMC3694829; doi:10.1371/journal.pgen.1003581)

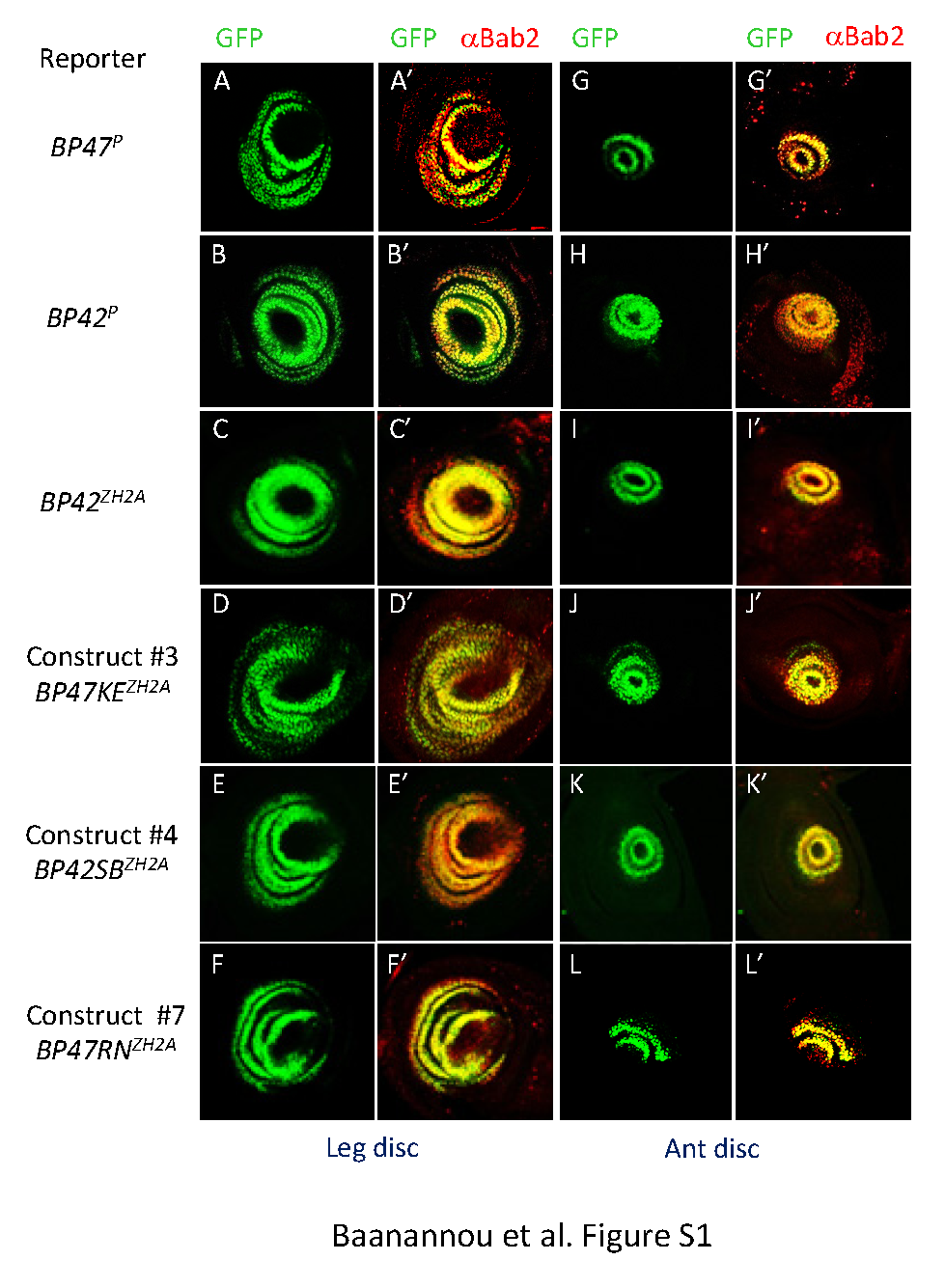

Supplement: Figure S1 — GFP-reporter constructs recapitulating leg and antennal bab2 expression. Leg (A–F) and antennal (ant) (G–L) imaginal discs from late third-instar larvae expressing GFP-reporter constructs (depicted in the left side) shown in Figure 1. GFP expression (green) alone and combined with Bab2 immunostaining (red) are shown for each construct (A–L and A′–L′, respectively). The reporter constructs encompassing the 1.5 kb-long BP47/42 overlapping region, including the 1.5 kb genomic fragment in isolation (construct #7), all faithfully drove limb-specific bab2 expression. (TIF) [file pgen.1003581.s001.tif]

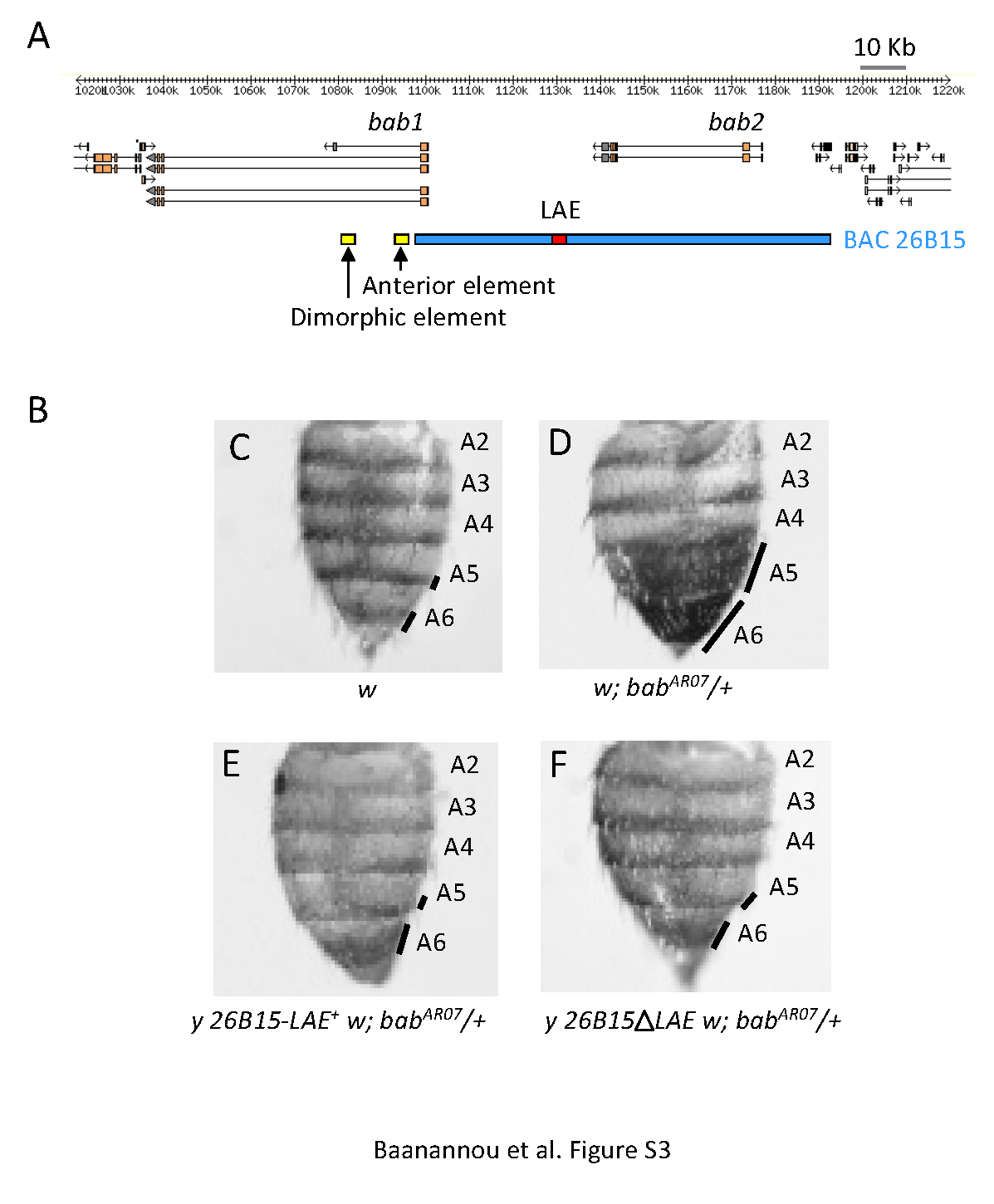

Supplement: Figure S3 — The 26B15 BAC partially rescues bab mutant abdominal phenotypes, independently of the LAE. (A) The 150-kb bab locus is shown (see Figure 1A). The position of the bab2-containing 26B15 BAC (in blue) used for phenotypic rescue experiments is indicated, with the internal LAE depicted as a red box. The positions of the two abdominal-specific cis-regulatory elements, within the bab1 transcription unit, are indicated as yellow boxes. (B) Dorsal views of female abdomens from the wild-type (C) and babAR07 heterozygous, carrying none (D) or a 26B15 BAC construct copy either unmodified (E) or LAE-deleted (F), are shown. Whereas pigmentation of wild-type abdominal tergites on segment A2–6 is limited to posterior stripes, females carrying a single babAR07 allele display nearly fully-pigmented A5–6 segments, evoking male-specific pigmentation. For the A5–6 segments of each genotype, pigmentation extends toward the anterior are indicated by solid bars. The bab mutant pigmentation defects are partially rescued in females carrying either an unmodified or a LAE-deleted 26B15 BAC construct. (TIF) [file pgen.1003581.s003.tif]

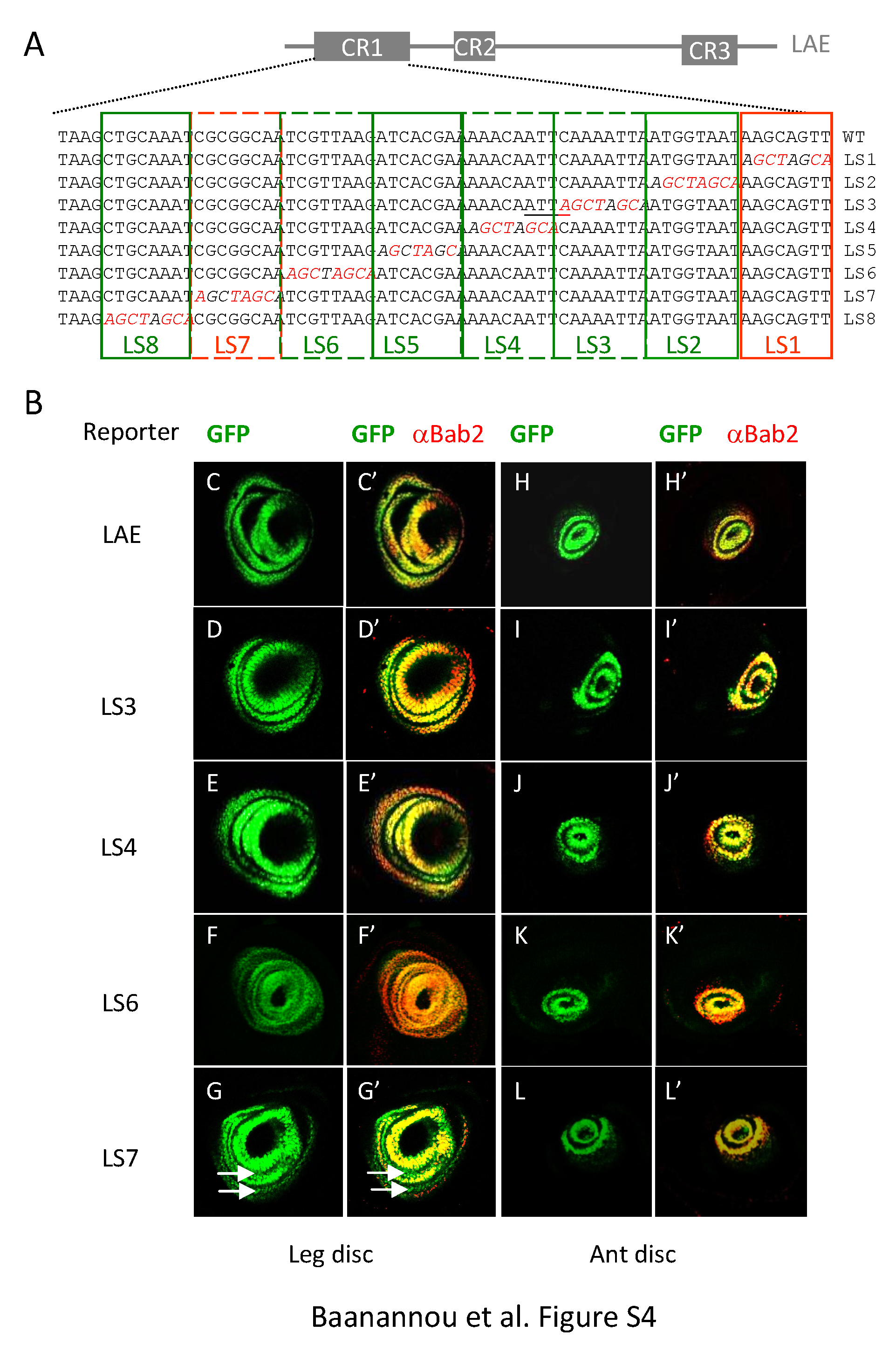

Supplement: Figure S4 — A linker-scanning mutagenesis of the critical CR1 region reveals functionally relevant motifs. (A) The sequences of the wild-type and of each mutated CR1 sub-region are shown in beneath the entire LAE structural organization, as determined by evolutionary conservation among Drosophilidae (Figure S2). For each of the eight mutated constructs, the inserted linker sequence (AGCTAGCA) is italicized with nucleotide substitutions depicted in red. Positively- or negatively-acting elements are framed in green or red, respectively, dashed lines indicating partially redundant functions. (B) Leg (C–G) and antennal (ant) (H–L) imaginal discs from late third-instar larvae, expressing either the unmodified LAE-GFP construct or one out of its four LS-mutated derivatives not shown in Figure 4. GFP fluorescence emission (green) in isolation and in combination with Bab2 immunostaining (red) (C–L and C′–L′, respectively), are shown. (TIF) [file pgen.1003581.s004.tif]

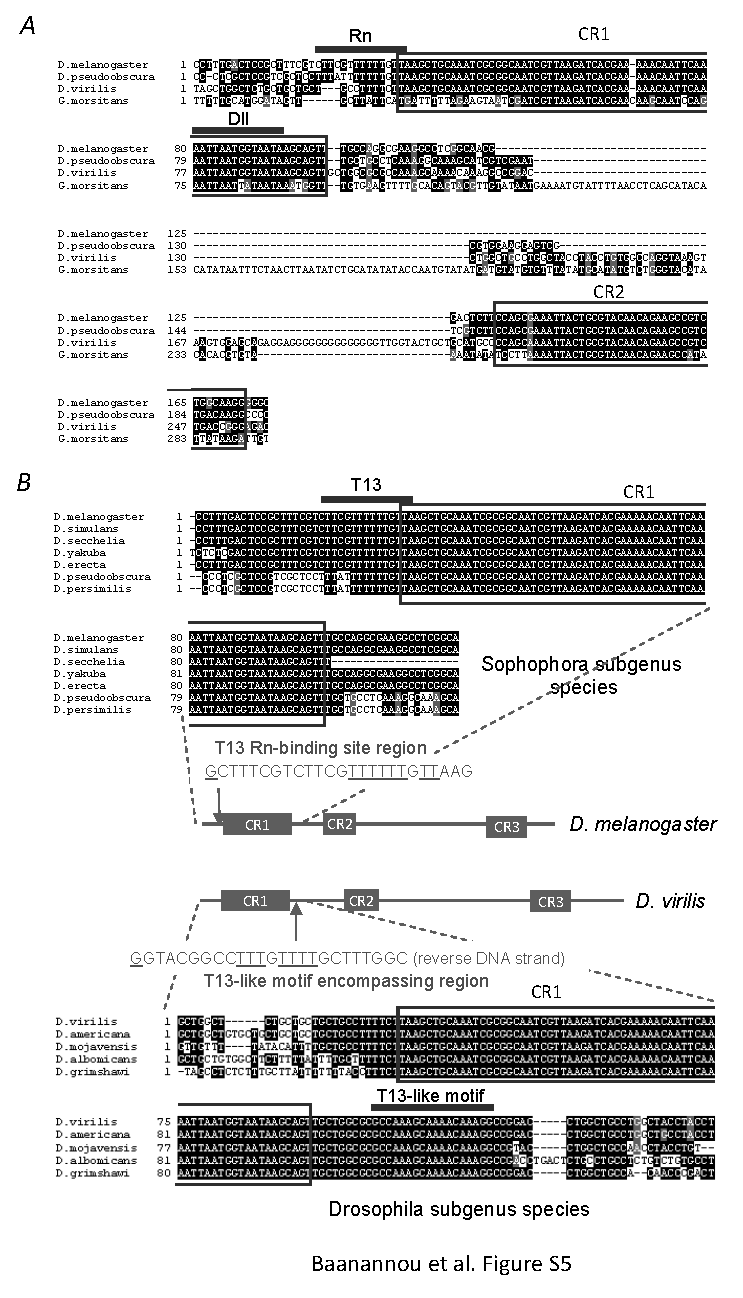

Supplement: Figure S5 — LAE sequences have been conserved among Dipterans. (A) Sequence conservation of the limb-specificity LAE portion among Drosophilidae and in the Glossinidae Glossina morsitans. G. morsitans LAE-like sequence was identified though blast analyses using the Trace archive nucleotide blast server at NCBI (http://blast.ncbi.nlm.nih.gov/Blast.cgi) and aligned with LAE sequences from representative Drosophilidae species, using MAFFT (http://mafft.cbrc.jp/alignment/server/index.html). Homology shading was made using BoxShade (http://www.ch.embnet.org/software/BOX_form.html). The CR1-2 sequences are framed and locations of the Rn and Dll binding sites are indicated above the alignment. Note that the Rn binding site is poorly conserved. (B) Sequence conservation of the CR1-encompassing LAE portion among Sophophora and Drosophila subgenera. Sequences were aligned and processed as above. The structural organization scheme of the entire D. melanogaster (Sophophora subgenus) and D. virilis (Drosophila subgenus) LAE sequences are shown in the middle part, with the aligned portions depicted with broken lines. The T13 Rn-binding site of D. melanogaster and the T13-like sequence of D. virilis are well conserved among Sophophora and Drosophila subgenus species, respectively (T-rich sequences are underlined). Note that the T13-like D. virilis sequence is located (i) in a 3′-end extended CR1 and (ii) in an inverted orientation. (TIF) [file pgen.1003581.s005.tif]
