# Supplementary material for: Drosophila Distal-less and Rotund Bind a Single Enhancer Ensuring Reliable and Robust bric-a-brac2 Expression in Distinct Limb Morphogenetic Fields
Source: PLoS Genet. 2013 Jun 27;9(6):e1003581. doi: 10.1371/journal.pgen.1003581 (PMC3694829; doi:10.1371/journal.pgen.1003581)
Supplement: Figure S2 — LAE sequence alignment from 22 Drosophila species. The genomic sequences homologous to the D. melanogaster LAE were recovered by Blat analysis at the UCSC Genome Browser website (http://genome.ucsc.edu/cgi-bin/hgBlat?command=start) and aligned with MAFFT (http://mafft.cbrc.jp/alignment/server/). The multiple alignment was then shaded with Boxshade (http://www.ch.embnet.org/software/BOX_form.html). The large (>20 bp) highly-conserved regions (CR1–3) are framed. Notice that D. mojavensis and D. willistoni LAE sequences have long inserts (1–2 kb) between the CR2 and CR3 regions. (PDF) [file pgen.1003581.s002.pdf]

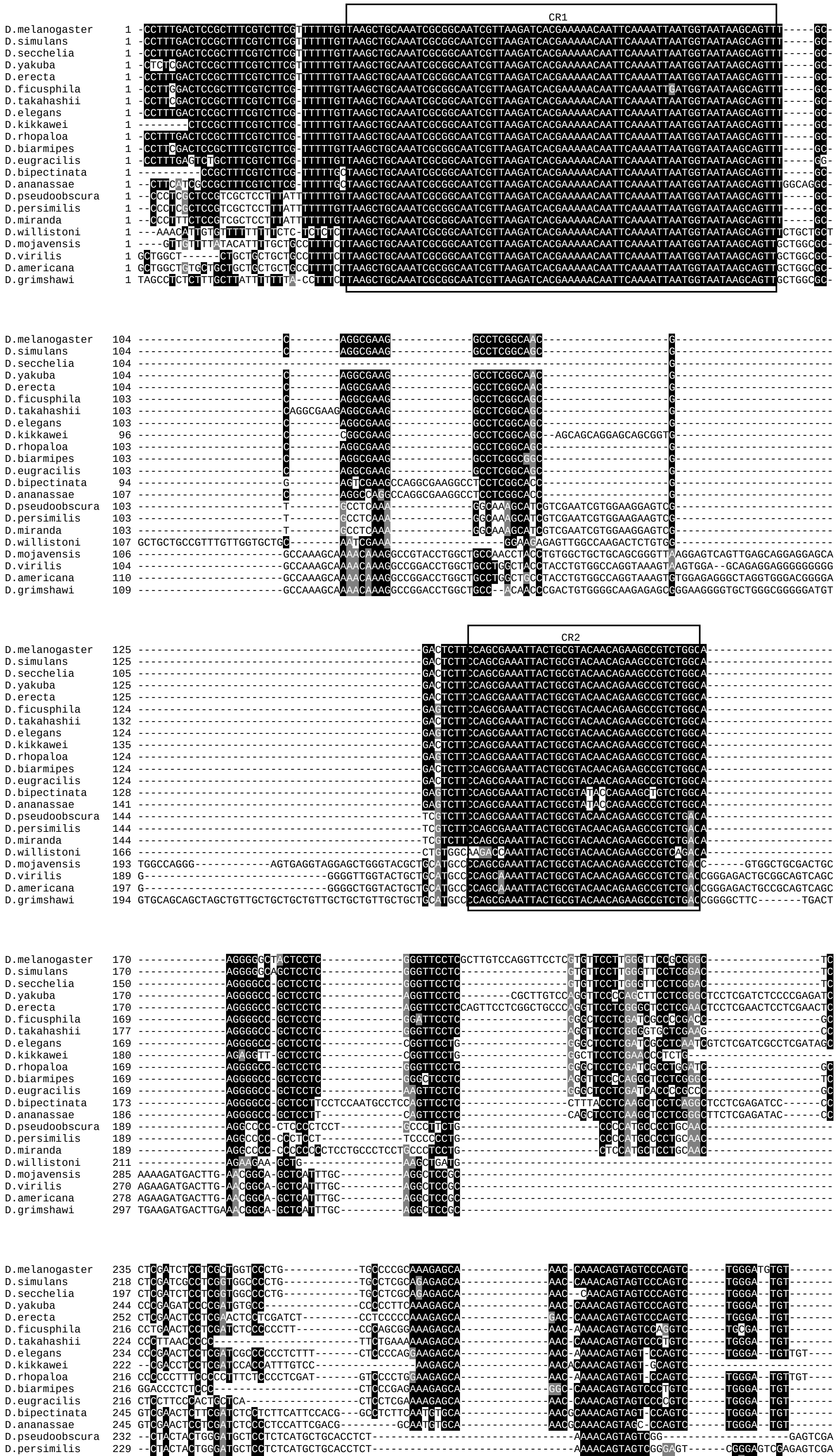

CR1
CR2
D.melanogaster
D.simulans
D.secchelia
D.yakuba
D.erecta
D.ficusphila
D.takahashii
D.elegans
D.kikkawei
D.rhopaloa
D.biarmipes
D.eugracilis
D.bipectinata
D.ananassae
D.pseudoobscura
D.persimilis
D.miranda
D.willistoni
D.mojavensis
D.virilis
D.americana
D.grimshawi

```
D.melanogaster  305 --------------------------------------------------------------------------------------------------------------
D.simulans      286 --------------------------------------------------------------------------------------------------------------
D.secchelia     264 --------------------------------------------------------------------------------------------------------------
D.yakuba        309 --------------------------------------------------------------------------------------------------------------
D.erecta        323 --------------------------------------------------------------------------------------------------------------
D.ficusphila    286 --------------------------------------------------------------------------------------------------------------
D.takahashii    281 --------------------------------------------------------------------------------------------------------------
D.elegans       309 --------------------------------------------------------------------------------------------------------------
D.kikkawei      276 --------------------------------------------------------------------------------------------------------------
D.rhopaloa      291 --------------------------------------------------------------------------------------------------------------
D.biarmipes     273 --------------------------------------------------------------------------------------------------------------
D.eugracilis    278 --------------------------------------------------------------------------------------------------------------
D.bipectinata   322 --------------------------------------------------------------------------------------------------------------
D.ananassae     316 --------------------------------------------------------------------------------------------------------------
D.pseudoobscura 288 GAGTCTGTG-----------------------------------------------------------------------------------------------------
D.persimilis    299 GAGTCTGTG-----------------------------------------------------------------------------------------------------
D.miranda       312 GAGTCTGTG-----------------------------------------------------------------------------------------------------
D.willistoni    316 --------------------------------------------------------------------------------------------------------------
D.mojavensis    370 GCGGCTGCTGCTGCTGCTGCTGCTTCTCCTCCTTCTTTTTGTATGCTTGCTCAAAAAGTTAAATGAGGCTATATTAGTTTTTAGTTTACTAAAGTTATGTGCGCTGAAAG
D.virilis       360 TTGTTGCCAGCTCT------GTTTTTTTTTATATCTCTTT----------------------------------------------------------------------
D.americana     368 TTGTTGCCAACTCTNNNNNNNNNTTTTTTTTTATATCTTT----------------------------------------------------------------------
D.grimshawi     389 TT---------------TGCTACCTTTTCTCTTTCCTCAC----------------------------------------------------------------------

D.melanogaster  305 --------------------------------------------------------------------------------------------------------------
D.simulans      286 --------------------------------------------------------------------------------------------------------------
D.secchelia     264 --------------------------------------------------------------------------------------------------------------
D.yakuba        309 --------------------------------------------------------------------------------------------------------------
D.erecta        323 --------------------------------------------------------------------------------------------------------------
D.ficusphila    286 --------------------------------------------------------------------------------------------------------------
D.takahashii    281 --------------------------------------------------------------------------------------------------------------
D.elegans       309 --------------------------------------------------------------------------------------------------------------
D.kikkawei      276 --------------------------------------------------------------------------------------------------------------
D.rhopaloa      291 --------------------------------------------------------------------------------------------------------------
D.biarmipes     273 --------------------------------------------------------------------------------------------------------------
D.eugracilis    278 --------------------------------------------------------------------------------------------------------------
D.bipectinata   322 --------------------------------------------------------------------------------------------------------------
D.ananassae     316 --------------------------------------------------------------------------------------------------------------
D.pseudoobscura 297 --------------------------------------------------------------------------------------------------------------
D.persimilis    308 --------------------------------------------------------------------------------------------------------------
D.miranda       321 --------------------------------------------------------------------------------------------------------------
D.willistoni    316 --------------------------------------------------------------------------------------------------------------
D.mojavensis    480 GATAGTTGAGTGAAGTTAACATCAAGAAATTTTATAAGTTGTTAAGAACTTTATAGCAAAAACTATAAGGATAAACTATAGAACTGTGTAAGGACTGGACAAAAATTCAC
D.virilis       394 --------------------------------------------------------------------------------------------------------------
D.americana     408 --------------------------------------------------------------------------------------------------------------
D.grimshawi     414 --------------------------------------------------------------------------------------------------------------

D.melanogaster  305 --------------------------------------------------------------------------------------------------------------
D.simulans      286 --------------------------------------------------------------------------------------------------------------
D.secchelia     264 --------------------------------------------------------------------------------------------------------------
D.yakuba        309 --------------------------------------------------------------------------------------------------------------
D.erecta        323 --------------------------------------------------------------------------------------------------------------
D.ficusphila    286 --------------------------------------------------------------------------------------------------------------
D.takahashii    281 --------------------------------------------------------------------------------------------------------------
D.elegans       309 --------------------------------------------------------------------------------------------------------------
D.kikkawei      276 --------------------------------------------------------------------------------------------------------------
D.rhopaloa      291 --------------------------------------------------------------------------------------------------------------
D.biarmipes     273 --------------------------------------------------------------------------------------------------------------
D.eugracilis    278 --------------------------------------------------------------------------------------------------------------
D.bipectinata   322 --------------------------------------------------------------------------------------------------------------
D.ananassae     316 --------------------------------------------------------------------------------------------------------------
D.pseudoobscura 297 --------------------------------------------------------------------------------------------------------------
D.persimilis    308 --------------------------------------------------------------------------------------------------------------
D.miranda       321 --------------------------------------------------------------------------------------------------------------
D.willistoni    316 --------------------------------------------------------------------------------------------------------------
D.mojavensis    590 TACGATATCTTGTACAAATTGTACATTATCGAAATTTGCATATATTTTATTGAATTTTAAATATATTCTTGGATAGACATACATATATTATCATATTATACTACTTACAG
D.virilis       394 --------------------------------------------------------------------------------------------------------------
D.americana     408 --------------------------------------------------------------------------------------------------------------
D.grimshawi     414 --------------------------------------------------------------------------------------------------------------

D.melanogaster  305 --------------------------------------------------------------------------------------------------------------
D.simulans      286 --------------------------------------------------------------------------------------------------------------
D.secchelia     264 --------------------------------------------------------------------------------------------------------------
D.yakuba        309 --------------------------------------------------------------------------------------------------------------
D.erecta        323 --------------------------------------------------------------------------------------------------------------
D.ficusphila    286 --------------------------------------------------------------------------------------------------------------
D.takahashii    281 --------------------------------------------------------------------------------------------------------------
D.elegans       309 --------------------------------------------------------------------------------------------------------------
D.kikkawei      276 --------------------------------------------------------------------------------------------------------------
D.rhopaloa      291 --------------------------------------------------------------------------------------------------------------
D.biarmipes     273 --------------------------------------------------------------------------------------------------------------
D.eugracilis    278 --------------------------------------------------------------------------------------------------------------
D.bipectinata   322 --------------------------------------------------------------------------------------------------------------
D.ananassae     316 --------------------------------------------------------------------------------------------------------------
D.pseudoobscura 297 --------------------------------------------------------------------------------------------------------------
D.persimilis    308 --------------------------------------------------------------------------------------------------------------
D.miranda       321 --------------------------------------------------------------------------------------------------------------
D.willistoni    316 --------------------------------------------------------------------------------------------------------------
D.mojavensis    700 TAAATGGTAAACTCATTCAATGAAATTATAGGATAGGATATACTATTCATTGGCAGATGAAATTTAGTAGAATAGAAGAAGGATCTATAGGTATGTTAAGATCATATTAA
D.virilis       394 --------------------------------------------------------------------------------------------------------------
D.americana     408 --------------------------------------------------------------------------------------------------------------
D.grimshawi     414 --------------------------------------------------------------------------------------------------------------

D.melanogaster  305 --------------------------------------------------------------------------------------------------------------
D.simulans      286 --------------------------------------------------------------------------------------------------------------
D.secchelia     264 --------------------------------------------------------------------------------------------------------------
D.yakuba        309 --------------------------------------------------------------------------------------------------------------
D.erecta        323 --------------------------------------------------------------------------------------------------------------
D.ficusphila    286 --------------------------------------------------------------------------------------------------------------
D.takahashii    281 --------------------------------------------------------------------------------------------------------------
D.elegans       309 --------------------------------------------------------------------------------------------------------------
D.kikkawei      276 --------------------------------------------------------------------------------------------------------------
D.rhopaloa      291 --------------------------------------------------------------------------------------------------------------
D.biarmipes     273 --------------------------------------------------------------------------------------------------------------
D.eugracilis    278 --------------------------------------------------------------------------------------------------------------
D.bipectinata   322 --------------------------------------------------------------------------------------------------------------
D.ananassae     316 --------------------------------------------------------------------------------------------------------------
D.pseudoobscura 297 --------------------------------------------------------------------------------------------------------------
D.persimilis    308 --------------------------------------------------------------------------------------------------------------
D.miranda       321 --------------------------------------------------------------------------------------------------------------
D.willistoni    316 --------------------------------------------------------------------------------------------------------------
D.mojavensis    810 GAGTACTACAGGTATTTTTATTTCTTAATCATATCATTAATATACTTAATAAACCTATATTTCCGACTCCTGTTCTATAAAATCTTATCTTAATTCTAATCACATTTTTA
D.virilis       394 --------------------------------------------------------------------------------------------------------------
D.americana     408 --------------------------------------------------------------------------------------------------------------
D.grimshawi     414 --------------------------------------------------------------------------------------------------------------
```

```
D.melanogaster   305 --------------------------------------------------------------------------------------------------------------
D.simulans       286 --------------------------------------------------------------------------------------------------------------
D.secchelia      264 --------------------------------------------------------------------------------------------------------------
D.yakuba         309 --------------------------------------------------------------------------------------------------------------
D.erecta         323 --------------------------------------------------------------------------------------------------------------
D.ficusphila     286 --------------------------------------------------------------------------------------------------------------
D.takahashii     281 --------------------------------------------------------------------------------------------------------------
D.elegans        309 --------------------------------------------------------------------------------------------------------------
D.kikkawei       276 --------------------------------------------------------------------------------------------------------------
D.rhopaloa       291 --------------------------------------------------------------------------------------------------------------
D.biarmipes      273 --------------------------------------------------------------------------------------------------------------
D.eugracilis     278 --------------------------------------------------------------------------------------------------------------
D.bipectinata    322 --------------------------------------------------------------------------------------------------------------
D.ananassae      316 --------------------------------------------------------------------------------------------------------------
D.pseudoobscura  297 --------------------------------------------------------------------------------------------------------------
D.persimilis     308 --------------------------------------------------------------------------------------------------------------
D.miranda        321 --------------------------------------------------------------------------------------------------------------
D.willistoni     316 --------------------------------------------------------------------------------------------------------------
D.mojavensis     920 AGTATTTTTATATGCCTATACAACCCAATTCAATCTGTTAATTTGTGCATTAACTCTAATATAGCTATACCTAATATACCTATTTTCCCGAGTTATGTTCTATAGAATC
D.virilis        394 --------------------------------------------------------------------------------------------------------------
D.americana      408 --------------------------------------------------------------------------------------------------------------
D.grimshawi      414 --------------------------------------------------------------------------------------------------------------

D.melanogaster   305 --------------------------------------------------------------------------------------------------------------
D.simulans       286 --------------------------------------------------------------------------------------------------------------
D.secchelia      264 --------------------------------------------------------------------------------------------------------------
D.yakuba         309 --------------------------------------------------------------------------------------------------------------
D.erecta         323 --------------------------------------------------------------------------------------------------------------
D.ficusphila     286 --------------------------------------------------------------------------------------------------------------
D.takahashii     281 --------------------------------------------------------------------------------------------------------------
D.elegans        309 --------------------------------------------------------------------------------------------------------------
D.kikkawei       276 --------------------------------------------------------------------------------------------------------------
D.rhopaloa       291 --------------------------------------------------------------------------------------------------------------
D.biarmipes      273 --------------------------------------------------------------------------------------------------------------
D.eugracilis     278 --------------------------------------------------------------------------------------------------------------
D.bipectinata    322 --------------------------------------------------------------------------------------------------------------
D.ananassae      316 --------------------------------------------------------------------------------------------------------------
D.pseudoobscura  297 --------------------------------------------------------------------------------------------------------------
D.persimilis     308 --------------------------------------------------------------------------------------------------------------
D.miranda        321 --------------------------------------------------------------------------------------------------------------
D.willistoni     316 --------------------------------------------------------------------------------------------------------------
D.mojavensis    1030 TTATCTTAAATCTAATCCTATTATTATCATATTATTAAATATTCTTTATAATCCTATGCATATCTGTTAATTTTTGTATTAAATCTAATATATCTATATTTCCGAGTTAT
D.virilis        394 --------------------------------------------------------------------------------------------------------------
D.americana      408 --------------------------------------------------------------------------------------------------------------
D.grimshawi      414 --------------------------------------------------------------------------------------------------------------

D.melanogaster   305 --------------------------------------------------------------------------------------------------------------
D.simulans       286 --------------------------------------------------------------------------------------------------------------
D.secchelia      264 --------------------------------------------------------------------------------------------------------------
D.yakuba         309 --------------------------------------------------------------------------------------------------------------
D.erecta         323 --------------------------------------------------------------------------------------------------------------
D.ficusphila     286 --------------------------------------------------------------------------------------------------------------
D.takahashii     281 --------------------------------------------------------------------------------------------------------------
D.elegans        309 --------------------------------------------------------------------------------------------------------------
D.kikkawei       276 --------------------------------------------------------------------------------------------------------------
D.rhopaloa       291 --------------------------------------------------------------------------------------------------------------
D.biarmipes      273 --------------------------------------------------------------------------------------------------------------
D.eugracilis     278 --------------------------------------------------------------------------------------------------------------
D.bipectinata    322 --------------------------------------------------------------------------------------------------------------
D.ananassae      316 --------------------------------------------------------------------------------------------------------------
D.pseudoobscura  297 --------------------------------------------------------------------------------------------------------------
D.persimilis     308 --------------------------------------------------------------------------------------------------------------
D.miranda        321 --------------------------------------------------------------------------------------------------------------
D.willistoni     316 --------------------------------------------------------------------------------------------------------------
D.mojavensis    1140 GTCCTTTTAAATCATACCTTTAAAGATCATCACAAAATGTTGTCCAGAACAAATTTCTCTATAGTTTTAGTTTCTCTTAAGCTTGAGCGGGTATTTCGTAGTCATTTTGA
D.virilis        394 --------------------------------------------------------------------------------------------------------------
D.americana      408 --------------------------------------------------------------------------------------------------------------
D.grimshawi      414 --------------------------------------------------------------------------------------------------------------

D.melanogaster   305 -------------------------------------------TGGGGCGTTCCAC---TCGCC--------GGCCTGGGACTCAAAACCCTCGGATGCCCCATGTCCG-
D.simulans       286 -------------------------------------------TGGGGCGTTCCAC---TCGCC--------GGCCTGGGACTCGAAACCCTCGGATGCCCCATGTCCG-
D.secchelia      264 -------------------------------------------TGGGGCGTTCCAC---TCGCC--------GGCCTGGGACTCGAAACCCTCGGATGCCCCATGTCCG-
D.yakuba         309 -------------------------------------------TGGGGCGTTCCAC---TCGCC--------GGCCTGGGACTCAAAGCCTCGGATGCCCCATGCTCCA-
D.erecta         323 -------------------------------------------TGGGGCGTTCCAC---TCGCC--------GGCCTGGGACTCAAAACCTCGGATGCCCCATGCCCC--
D.ficusphila     286 -------------------------------------------TGGGGCGTTCCACAT-TTGCC--------GGCCTGGGACTCAAACTCGGGCCGAAACTCTGCCCTC-
D.takahashii     281 -------------------------------------------TGGGGCGTTTCAC---TCGCCGG------GGCCTGGGACTCAGACTCCAACTCAAAGCGAACTCCG-
D.elegans        309 -------------------------------------------CGGGGCGTTTCAC---TCGCC--------GGCCTGGGACTCAAACTCAAAGTCAAACTCTGAAACT-
D.kikkawei       276 -------------------------------------------TGGGGCGTTTCAC---TCCAT--------GGCCTGGGACTCCAGAGACCAGACCGAGGCTCTGCCC-
D.rhopaloa       291 -------------------------------------------TGGGGCGTTCCAC---TCACC--------GGCCTGGGACTCAAACTCAAACTCAAACTGAAGCCCT-
D.biarmipes      273 -------------------------------------------TGGGGCGTTCCAC---TCGCC--------GGCCTGGGACTCAAGCTCGCAACGAACTCCGACCAAC-
D.eugracilis     278 -------------------------------------------TGGGGCGTTCCACTGCTCGCT--------GGCCTGGGACTCAAAGCTCCGACAGCGTCCAACTCTC-
D.bipectinata    322 -------------------------------------------TGGGGCGTTCCAT---CCGAT--------GGCCTGAGACTCGGGACTCCAACTCTGCCCTCGTCCA-
D.ananassae      316 -------------------------------------------TGGGGCGTTCCAT---CCGAT--------GGCCTGGGACTTGGGACTCCAACTCTGCCCTCGTCCA-
D.pseudoobscura  297 --------------------------------------GCATGGAGGGCGTTCCACTG-GCGCTGAGACTTTTGCCTGAGAGCCGCTG----------------------
D.persimilis     308 --------------------------------------GCATGGGGGGCGTTCCACTG-GCGCTGAGACTTTTGCCTGAGAGCCGCTG----------------------
D.miranda        321 --------------------------------------GCATGGGGGGCGTTCCACTG-GCGCTGAGACTTTTGCCTGAGAGCCGCTG----------------------
D.willistoni     316 ---------------------------------------------GGGCGTGGCAGATGGCGCTGCCAGAG-AGACTGAGATGTAGAC----------------------
D.mojavensis    1250 TCTCACTGCCATGCTCTTCCTCGTTTTTTTGCTAATTTTTTTTCGGGGCGTTGCGCCG-GCGCT---------GCGCAGGAGCCAGAG----------------------
D.virilis        394 --------------------------------------TTTTTCGGGGCGTTGCGCCG-GCGCTGAGTGGCAAACCCAGGAGCTGAAG----------------CTGGAA
D.americana      408 --------------------------------------TTTTTCGGGGCGTTGCGCCG-GCGCTGAGTGGCAAACCCAGGAGCTGAAG----------------CTGGA-
D.grimshawi      414 --------------------------------------TTTTTTTGGGCGTTGCGCTG-GCGCTGAGC--------------CTGGAC----------------CTGGTC

D.melanogaster   360 ---------------------------ATGCCAGATTCTCGGATCG-----TTGATCGATGG------------A---------TGCTGC--------------------
D.simulans       341 ---------------------------TTGCCAGATTCTCGGATCG-----TTGATCGATGG------------A---------TGCTGC--------------------
D.secchelia      319 ---------------------------ATGCCAGATTCTCGGATCG-----TTGATCGATGG------------A---------TGCTGC--------------------
D.yakuba         364 TGCTCCATGCTCCATGCCCCATGCCACATGCCCGATTCTCGGATCG-----TTGATCGATGG------------A---------TGCTGC--------------------
D.erecta         377 ----------------------------------ATTCTCGGATCG-----TTGATCGATGG------------A---------TGCTGC--------------------
D.ficusphila     343 ----------------------------------ATCCTCGGATCT-----CTGATCGATGG------------A---------TGCTGC--------------------
D.takahashii     338 ------AATAAAACTCTGCCTCGGTTGCCTTTGGATCCTCGGATCG-----TTGATCGATGG------------A---------TGCTGC--------------------
D.elegans        364 ------------------CTGAAACTCTGCCCTCGTCCTCAGATCG-----TTGATCGATGG------------A---------TGCTGC--------------------
D.kikkawei       331 ---------------------TCATCCTTACATGGACTCCAGATCG-----TTGATCGATGG------------A---------TGCTGC--------------------
D.rhopaloa       346 -------------------------CATCCTCGGATCCTCGGATCG-----TTGATCGATGG------------A---------TGCTGC--------------------
D.biarmipes      328 --------------------------TCTGGACGATCTTCGGATCG-----TTGATCGATGG------------A---------CGCTGC--------------------
D.eugracilis     336 -------------------------GGACCGTCGATCGACGGATGGATGGATGGATGGATGG------------A---------TGCTGC--------------------
D.bipectinata    377 ---------------------------AGCAGCCACTGTCAGATCG-----CTGATCGATGG------------A---------TGCTGC--------------------
D.ananassae      371 ---------------------------TGCAGCCTCTGTCAGATCG-----CTGATCGATGG------------A---------TGCTGC--------------------
D.pseudoobscura  346 ---------------------------------------------C-----TTGATCGATGG------------A---------TGCTGCCACTGCTGCCACTGCCACTG
D.persimilis     357 ---------------------------------------------C-----TTGATCGATGG------------ATGCTGCCACTGCTGCCACTGCTGCCACTGCCACTG
D.miranda        370 ---------------------------------------------C-----TTGATCGATGG------------A---------TGCTGCCACTG------CTGCCACTG
D.willistoni     358 ----------------------TTCTTCATTTTTAGTCTTCGATCG-----TTGATCGATGG------------------------CTGC--------------------
D.mojavensis    1328 ------------------------------------CTGTAGACCCCAGCGTTGATCGATGGCGGC--------CTCTCGCTTCTGCTGCTGCTGCTGCTGCTGCTGCTG
D.virilis        449 CTGGAGCTGGAGAAGGAGCAGCAGCAGAAGACGCGGCCG------------TTGATCGATGGCTTCTTCTGCTGCGTCTGCTTCTGCTGC--------------------
D.americana      462 --------NNNNNNNNNGCAGCCGCAGAAGACGCGGCCG------------TTGATCGATGGCTTCTTCAGCTGCTTCTGCTTCTGCTGC--------------------
D.grimshawi      455 GTGGAGCTGGA-------------CAGAAGCCACGGCCTCGGAAAG-----TTGATCGATCG------------------------CTGT--------------------
```

```
D.melanogaster   397 ----------------TGGC------------------------------------------------------------------------------------------
D.simulans       378 ----------------TGGC------------------------------------------------------------------------------------------
D.secchelia      356 ----------------TGGC------------------------------------------------------------------------------------------
D.yakuba         428 ----------------TGGC------------------------------------------------------------------------------------------
D.erecta         407 ----------------TGGC------------------------------------------------------------------------------------------
D.ficusphila     373 ----------------TGGC------------------------------------------------------------------------------------------
D.takahashii     396 ----------------TGGC------------------------------------------------------------------------------------------
D.elegans        410 ----------------TGGC------------------------------------------------------------------------------------------
D.kikkawei       374 ----------------TGGC------------------------------------------------------------------------------------------
D.rhopaloa       385 ----------------TGGC------------------------------------------------------------------------------------------
D.biarmipes      366 ----------------TGGC------------------------------------------------------------------------------------------
D.eugracilis     380 ----------------TGGC------------------------------------------------------------------------------------------
D.bipectinata    414 ----------------TGGC------------------------------------------------------------------------------------------
D.ananassae      408 ----------------TGGC------------------------------------------------------------------------------------------
D.pseudoobscura  385 CCACTGCTGCTGCTTGTGGC------------------------------------------------------------------------------------------
D.persimilis     405 CCACTGCTGCTGCTTGTGGC------------------------------------------------------------------------------------------
D.miranda        403 CCACTGCTGCTGCTTGTGGC------------------------------------------------------------------------------------------
D.willistoni     396 ----------------TGGCTACCAACTGCACTCCCCGTCTACCCTTTCCCCATTCTCCTTACCTTGTATCTGTACATTTTTTTCTTATACCCATTAAGCTTAGAGATGA
D.mojavensis    1394 C---------------TGGC------------------------------------------------------------------------------------------
D.virilis        527 ----------------TGGC------------------------------------------------------------------------------------------
D.americana      532 ----------------TGGC------------------------------------------------------------------------------------------
D.grimshawi      503 ----------------TGGC------------------------------------------------------------------------------------------

D.melanogaster   401 --------------------------------------------------------------------------------------------------------------
D.simulans       382 --------------------------------------------------------------------------------------------------------------
D.secchelia      360 --------------------------------------------------------------------------------------------------------------
D.yakuba         432 --------------------------------------------------------------------------------------------------------------
D.erecta         411 --------------------------------------------------------------------------------------------------------------
D.ficusphila     377 --------------------------------------------------------------------------------------------------------------
D.takahashii     400 --------------------------------------------------------------------------------------------------------------
D.elegans        414 --------------------------------------------------------------------------------------------------------------
D.kikkawei       378 --------------------------------------------------------------------------------------------------------------
D.rhopaloa       389 --------------------------------------------------------------------------------------------------------------
D.biarmipes      370 --------------------------------------------------------------------------------------------------------------
D.eugracilis     384 --------------------------------------------------------------------------------------------------------------
D.bipectinata    418 --------------------------------------------------------------------------------------------------------------
D.ananassae      412 --------------------------------------------------------------------------------------------------------------
D.pseudoobscura  405 --------------------------------------------------------------------------------------------------------------
D.persimilis     425 --------------------------------------------------------------------------------------------------------------
D.miranda        423 --------------------------------------------------------------------------------------------------------------
D.willistoni     490 AGGGTATATTAAGATCTCATAAAAATTAATAAATTCCCTATTGTCCATCTAAATGTTAGAGCTATCAAATTTTGCTGGGAAGACGCTTATTAACTGGTTTGTTACTTTAG
D.mojavensis    1399 --------------------------------------------------------------------------------------------------------------
D.virilis        531 --------------------------------------------------------------------------------------------------------------
D.americana      536 --------------------------------------------------------------------------------------------------------------
D.grimshawi      507 --------------------------------------------------------------------------------------------------------------

D.melanogaster   401 --------------------------------------------------------------------------------------------------------------
D.simulans       382 --------------------------------------------------------------------------------------------------------------
D.secchelia      360 --------------------------------------------------------------------------------------------------------------
D.yakuba         432 --------------------------------------------------------------------------------------------------------------
D.erecta         411 --------------------------------------------------------------------------------------------------------------
D.ficusphila     377 --------------------------------------------------------------------------------------------------------------
D.takahashii     400 --------------------------------------------------------------------------------------------------------------
D.elegans        414 --------------------------------------------------------------------------------------------------------------
D.kikkawei       378 --------------------------------------------------------------------------------------------------------------
D.rhopaloa       389 --------------------------------------------------------------------------------------------------------------
D.biarmipes      370 --------------------------------------------------------------------------------------------------------------
D.eugracilis     384 --------------------------------------------------------------------------------------------------------------
D.bipectinata    418 --------------------------------------------------------------------------------------------------------------
D.ananassae      412 --------------------------------------------------------------------------------------------------------------
D.pseudoobscura  405 --------------------------------------------------------------------------------------------------------------
D.persimilis     425 --------------------------------------------------------------------------------------------------------------
D.miranda        423 --------------------------------------------------------------------------------------------------------------
D.willistoni     600 AAAATAAGCATTACTTTACAAAAAGCATTACTTTTGCAGTATTTAAGGTAATTAAACACCTCAAATGACTAATTATGGATTACATTTCTTAAATTTAAGCGTCAGAACCG
D.mojavensis    1399 --------------------------------------------------------------------------------------------------------------
D.virilis        531 --------------------------------------------------------------------------------------------------------------
D.americana      536 --------------------------------------------------------------------------------------------------------------
D.grimshawi      507 --------------------------------------------------------------------------------------------------------------

D.melanogaster   401 --------------------------------------------------------------------------------------------------------------
D.simulans       382 --------------------------------------------------------------------------------------------------------------
D.secchelia      360 --------------------------------------------------------------------------------------------------------------
D.yakuba         432 --------------------------------------------------------------------------------------------------------------
D.erecta         411 --------------------------------------------------------------------------------------------------------------
D.ficusphila     377 --------------------------------------------------------------------------------------------------------------
D.takahashii     400 --------------------------------------------------------------------------------------------------------------
D.elegans        414 --------------------------------------------------------------------------------------------------------------
D.kikkawei       378 --------------------------------------------------------------------------------------------------------------
D.rhopaloa       389 --------------------------------------------------------------------------------------------------------------
D.biarmipes      370 --------------------------------------------------------------------------------------------------------------
D.eugracilis     384 --------------------------------------------------------------------------------------------------------------
D.bipectinata    418 --------------------------------------------------------------------------------------------------------------
D.ananassae      412 --------------------------------------------------------------------------------------------------------------
D.pseudoobscura  405 --------------------------------------------------------------------------------------------------------------
D.persimilis     425 --------------------------------------------------------------------------------------------------------------
D.miranda        423 --------------------------------------------------------------------------------------------------------------
D.willistoni     710 CTAAAGTCCTGCAAATCCTGACAATGTGAATTTTGAAGTACAATTCTTCTTTTATTTTGCAGGGTATAAGCAAGTCGTAGCTGCAATTACTTACTTTATTGTGTTTTTTT
D.mojavensis    1399 --------------------------------------------------------------------------------------------------------------
D.virilis        531 --------------------------------------------------------------------------------------------------------------
D.americana      536 --------------------------------------------------------------------------------------------------------------
D.grimshawi      507 --------------------------------------------------------------------------------------------------------------

D.melanogaster   401 ------------------GTC-TTTTC-----GTTT---------------------------T--TGTTAGAGCAGCAGAAAGCAGAAAGCTGGGGGAGC--------A
D.simulans       382 ------------------GTC-TTTTC-----GTTT---------------------------T--TGTTAGAGCAGCAGAAAGCAGAAAGCTGGGG------------A
D.secchelia      360 ------------------GTC-TTTTC-----GTTT---------------------------T--TGTTAGAGCAGCAGAAAGCAGAAAGCTGGGGG-----------A
D.yakuba         432 ------------------GTC-TTTTC-----GTTT---------------------------T--TGTTAGAGG----------------------------------A
D.erecta         411 ------------------GTC-TTTTC-----GTTT---------------------------T--TGTTAGAGCAGCGGAAAGCTGGGG-------------------A
D.ficusphila     377 ------------------GTC-TTTTC-----TTTT---------------------------T--TATTAGAGCCACGGCAGCAGCAACGGAAAAAAGCCGCAGT---A
D.takahashii     400 ------------------GTC-TTTTA-----TTTT---------------------------TTGTGTTAGAGCAACCAAGGCAAAGCAAAGCAAAGA----------A
D.elegans        414 ------------------GTTGTTTCT-----TTTT---------------------------T--TGTTAGAGCCACCGCAGC-------------------------A
D.kikkawei       378 ------------------GTC-TTTTC-----TTTTCTTCTCCGTTTTTTCTTTTTCTTTTTTTTGTGTTAGAGCAGCAGAAG---------------------------
D.rhopaloa       389 ------------------GTC-TTTTC-----TTTT---------------------------T--TGTTAGAGGAGCCAC----------------------------T
D.biarmipes      370 ------------------GTC-TTTTG-----CTTT---------------------------T--TGTTAGAGCAGCAGAGGCCGGGCCGAGA---------------A
D.eugracilis     384 ------------------GTC-TTTTC-----TTTT------------------------------TGTTAGAGCAGCAGAAAGCTGGAAG------------------A
D.bipectinata    418 ------------------GTC-CTTCC-----TTTT---------------------------TTTCGTTAGAGCTGAAAAAGCTGAAAAAA-----------------G
D.ananassae      412 ------------------GTC-TTCCCGTTTTTTTT---------------------------TTTCGTTAGAGCTGAAAAAGCTGAAAAAA-----------------G
D.pseudoobscura  405 ------------------ATCGTTTTT-----TTTTTTTGTTTTTTGTTTTTTGTCTGTTAGATGTTGCAGGAGTCTCGGA----------------------------G
D.persimilis     425 ------------------ATCGTTTTT-------TTTTTGTTTTTTGTTTTTTGTCTGTTAGATGTTGCAGGAGTCTCGGA----------------------------G
D.miranda        423 ------------------ATCGTTTTT-----T-------GTTTTTGTTTTTTGTCTGTTAGATGTTGCAGGAGTCTCGGA----------------------------G
D.willistoni     820 CTCTTTATTTAATTTTTTGTTGTATTTTTAGTTTTT----TTTTTTTTTTTTTTTGTTTTTGTTTCTGTTAGAA----------------------------GTCGTGTG
D.mojavensis    1399 ----------------------TATCTGCCTGGCATTTTTTTTATTTCTTATTTTTTTCGAGCTGCTGTTAGAA-------------------------GTCGTGTCGTG
D.virilis        531 ----------------------TATCTACCGGGTTTTTTTTCCATAT----TTTTTTTCGAGCTGCTGTTAGAA-------------------------GTCGTGTCGTG
D.americana      536 ----------------------TATCTGTT--TTTTTTTTTTCATAT----TTTTTTTCGAGCTGCTGTTAGAA-------------------------GTCGTGTCGTG
D.grimshawi      507 ----------------------AAACT-TTAGTTTCTTTTGTTATGT----TTT---------TGTTGTTAGAA-------------------------GTCGTGTCGTG
```

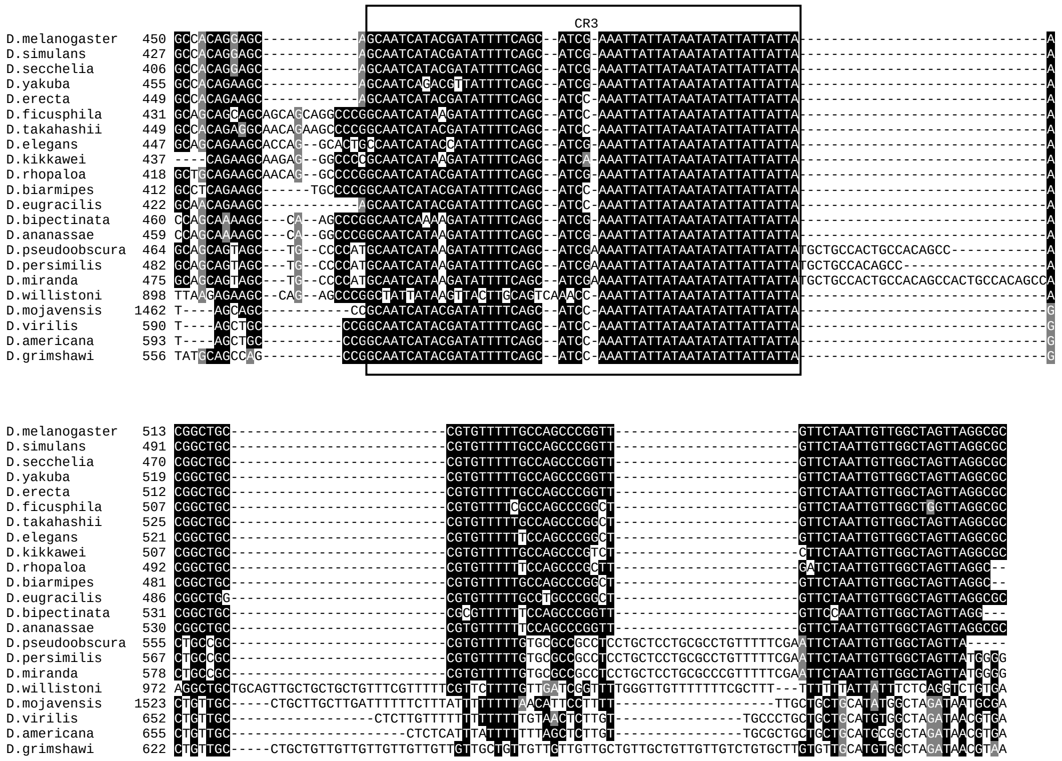

Figure S2
